# Study Design, Reconstructive Modalities, and Patient-Reported Outcome Documentation in a Curated Corpus of Facial Basal Cell Carcinoma Reconstruction Studies: An Exploratory Meta-Research Analysis

**DOI:** 10.3390/diagnostics16182952

**Published:** 2026-09-12

**Authors:** Iris-Iuliana Adam, Ruxandra-Cristina Marin, Mihnea Costescu, Liliana Vecerzan, Bogdan Moldovan, Ioana Roșca, Oana Andreia Coman, Raluca-Gabriela Miulescu, Ana-Maria Popa, Alexandru-Petru Ciucu, Alina Ormenișan

**Affiliations:** 1 St. Constantin Hospital, 500299 Brașov, Romania; adamirisiuliana@gmail.com (I.-I.A.); bogdan.moldovan@spitalulsfconstantin.ro (B.M.); miulescuraluca@yahoo.com (R.-G.M.); 2 Dental Elite Clinics, 500187 Brașov, Romania; alexandru.ciucu@dentalelite.ro; 3 Faculty of Medicine, “Carol Davila” University of Medicine and Pharmacy, 020021 Bucharest, Romania; ruxandra.marin@umfcd.ro (R.-C.M.); ioana.rosca@umfcd.ro (I.R.); oana.coman@umfcd.ro (O.A.C.);; 4 Discipline of Pharmacology, Clinical Pharmacology and Pharmacotherapy, “Carol Davila” University of Medicine and Pharmacy, 020021 Bucharest, Romania; 5 Faculty of Medicine, Lucian Blaga University of Sibiu, 550169 Sibiu, Romania; 6 Dr. Alexandru Augustin Military Emergency Clinical Hospital, 550024 Sibiu, Romania; 7 Faculty of Dental Medicine, George Emil Palade University of Medicine, Pharmacy, Science, and Technology of Târgu Mureș, 540142 Târgu Mureș, Romania; alina.ormenisan@umfst.ro; 8 Emergency Clinical County Hospital Târgu Mureș, 540136 Târgu Mureș, Romania

**Keywords:** basal cell carcinoma, facial reconstruction, curated evidence corpus, patient-reported outcomes, patient-reported outcome measures, meta-research, exploratory analysis

## Abstract

**Background/Objectives:** This exploratory meta-research study evaluated study design, reconstructive modalities, and outcome documentation within a non-systematically assembled corpus of facial basal cell carcinoma (BCC) reconstruction studies. The corpus was curated for a parent project, coding relied on narrative matrix fields, and no duplicate full-text extraction was available; frequencies should therefore be interpreted as minimum documentation estimates within this corpus. **Methods:** We analyzed a frozen matrix of 72 clinical studies with final issue years from 2015 through 2026 (analytic cutoff, 31 July 2026). Prespecified coding distinguished BCC-only from mixed-histology studies and separated any patient-reported endpoint, validated patient-reported outcome measures (PROMs), nonvalidated satisfaction assessments, observer-rated measures, and mixed patient/observer instruments. Associations with prospective design were exploratory. **Results:** Fifty-nine studies (81.9%) were retrospective, seven (9.7%) comparative, and three (4.2%) multicenter; forty (55.6%) were coded BCC-only. Local or locoregional flaps appeared in 48 matrix records (66.7%). Aesthetic outcomes were documented in 47 (65.3%), complications in 45 (62.5%), function in 27 (37.5%), and any patient-reported endpoint in 9 (12.5%). Four records (5.6%) documented a validated PROM; five (6.9%) named any standardized instrument, including one observer-only VSS assessment. Prospective studies more often documented patient-reported endpoints (4/9 vs. 5/63; OR 9.28, 95% CI 1.87–46.01; *p* = 0.011) and validated PROMs (3/9 vs. 1/63; OR 31.00, 95% CI 2.77–346.32; *p* = 0.005), but event counts were sparse and estimates were unadjusted. No matrix record documented all five prespecified outcome domains. **Conclusions:** Within this curated corpus, retrospective and noncomparative designs predominated and validated patient-centered measurement was uncommon. These findings are hypothesis-generating, do not estimate comparative effectiveness, and should not be generalized to the complete facial BCC reconstruction literature. A reproducible systematic review with duplicate full-text extraction remains necessary.

## 1. Introduction

Basal cell carcinoma is the most common cutaneous malignancy and arises disproportionately on chronically sun-exposed facial skin. Mortality is uncommon, but locally destructive disease and its treatment can affect eyelid competence, nasal airflow, oral continence, facial symmetry, scar perception, and psychosocial well-being. Contemporary European and British guidance therefore prioritizes complete surgery, risk-adapted margin control, and tissue preservation, particularly for high-risk or recurrent lesions on critical anatomical sites [1,2].

Clinicopathologic variables such as tumor size, anatomical location, histologic subtype, recurrence status, and margin-control strategy influence treatment and reconstructive planning [1,2]. The source matrix, however, did not contain sufficiently uniform full-text detail to support a dedicated quantitative analysis of diagnostic reporting. Diagnostic reporting was therefore removed from the title, and is discussed only as a clinical context and a future reporting priority.

Reconstruction after facial BCC excision is determined by tumor biology, margin-control strategy, defect geometry, facial aesthetic subunits, lamellar anatomy, vascularity, comorbidity, frailty, and patient expectations. Primary closure, skin grafting, local and regional flaps, free tissue transfer, secondary intention healing, and biomaterial-based approaches are all legitimate in selected settings. Mohs micrographic surgery may reduce unnecessary tissue sacrifice in infiltrative or high-risk facial BCC [3,4,5], but the final reconstructive choice remains strongly dependent on defect characteristics and local expertise.

Most published series describe favorable flap survival, low recurrence, or acceptable appearance. Such observations are clinically useful but do not necessarily establish comparative effectiveness. Technique-focused case series, mixed BCC and squamous cell carcinoma cohorts, short follow-up, subjective aesthetic judgments, and nonstandard outcome definitions can create a literature that is abundant yet difficult to synthesize. In facial oncology, success extends beyond margin clearance and includes function, appearance, treatment burden, quality of life, and the patient’s own appraisal of the result [6,7,8,9,10,11,12,13].

Patient-reported outcomes (PROs) are outcomes obtained directly from patients, whereas patient-reported outcome measures (PROMs) are the instruments used to measure them. FACE-Q and the Skin Cancer Index are PROMs [9,10]; POSAS combines patient and observer components [7]; and VSS is primarily an observer-rated scar scale [8]. A recent systematic review of PROMs after Mohs reconstruction found no consistently adopted instrument [14]. We therefore separated these constructs rather than treating every named scale as a PROM.

We performed an exploratory meta-research analysis of a frozen, curated matrix of 72 clinical studies addressing reconstruction after facial BCC or BCC-containing facial skin cancer excision. The objectives were to describe study architecture, reconstructive modalities, outcome documentation, and limitations recorded in the matrix; to distinguish BCC-only from mixed-histology evidence; and to examine exploratory associations between prospective design and patient-centered documentation. All inferences are restricted to the curated corpus.

## 2. Methods

### 2.1. Study Design and Reporting Framework

This investigation was designed as meta-research—a study-level, cross-sectional secondary analysis of a previously curated evidence matrix. Meta-research evaluates research methods, reporting, reproducibility, and research practices [15]. The individual report was the unit of analysis. Applicable STROBE items guided reporting of the cross-sectional study-level analysis [16]. We used only the SWiM items concerning transparent grouping, tabulation, and the discussion of synthesis limitations [17]; we do not claim SWiM compliance because this was not a systematic review. The original literature search and screening process was unavailable, and exhaustive coverage is not claimed. A STROBE-oriented map is provided in Supplementary Table S5.

### 2.2. Source Dataset and Analytic Eligibility

The source dataset was a narrative evidence matrix assembled for a parent project on facial BCC reconstruction and frozen for this analysis on 31 July 2026. It contained 72 nonempty records with final journal issue years from 2015 to 2026 and summarized author/year, country, design and sample size, tumor and facial site, reconstruction, predictors, outcomes, key results, and stated limitations. The original databases, search strings, duplicate-removal process, language restrictions, screening decisions, and full-text exclusion reasons were unavailable. Analytic eligibility was therefore operationalized within the supplied matrix; a record had to concern facial or head-and-neck reconstruction after the excision of BCC or a mixed malignant skin tumor cohort containing BCC. All nonempty eligible matrix records were retained. This denominator is a curated corpus, not a systematically identified sample of the field.

A record-by-record reconciliation linked every matrix row to its complete bibliographic reference, supplementary reference number, main text reference number, histologic description, anatomical description, eligibility status, and study-level analytic contribution. Six unrelated publications had been inserted accidentally into the main bibliography but were absent from Supplementary Table S1 and from the analytic matrix; they therefore made no contribution to any result. After their removal, the 72 corpus reports map exactly to main references [3,4,5,6,18,19,20,21,22,23,24,25,26,27,28,29,30,31,32,33,34,35,36,37,38,39,40,41,42,43,44,45,46,47,48,49,50,51,52,53,54,55,56,57,58,59,60,61,62,63,64,65,66,67,68,69,70,71,72,73,74,75,76,77,78,79,80,81,82,83,84,85] and Supplementary References S1–S72. Supplementary Table S6 documents this reconciliation.

### 2.3. Coding and Operational Definitions

A prespecified codebook was applied to the narrative matrix in two passes; structured tags were generated from the supplied fields and each row was then re-audited against the matrix. Coding was performed by one investigator; no independent duplicate full-text coder was available, and inter-rater agreement could not be estimated. Accordingly, a domain coded as absent means only that it was not documented in the matrix summary; it must not be interpreted as absent from the original publication. Definitions are provided in Supplementary Table S2.

- Study architecture: retrospective, prospective, comparative, case-series, multicenter, database-based, and cohort features.
- Histologic scope: BCC-only versus mixed histology or broader non-melanoma skin cancer.
- Anatomical focus: general face/head-and-neck, periocular/eyelid, nasal, other focal facial site, or unclear.
- Reconstructive modalities: primary/direct closure, local or locoregional flap, skin graft, free flap, secondary intention healing, biomaterial/allograft, and composite lamellar/cartilage/mucosal support. Categories were non-mutually exclusive.
- Outcome documentation: oncologic control, margin/tissue-sparing outcomes, complications/flap viability, aesthetics, function, and any patient-reported endpoint.
- Patient-centered measurement taxonomy: validated PROM; nonvalidated patient satisfaction assessment; validated clinician/observer-rated measure; and mixed patient/observer instrument. Any named standardized instrument was retained as a separate, broader category.
- Exploratory documentation breadth: documentation in the matrix of at least four of five domains—oncologic control, complications, aesthetics, function, and patient perspective.

### 2.4. Sample-Size Handling

When patient and lesion counts were both available, the patient count was preferred. When only lesions, tumors, cases, or excisions were recorded, that analytic unit was retained. Because these units are incompatible and one database record contained 96,458 patients [50], no summed denominator is emphasized or interpreted as a clinical cohort. Study size is summarized by the median and interquartile range. Exclusion of the database record was retained only as a denominator-influence check; the principal study-level analyses were unweighted, so little change was expected.

### 2.5. Outcomes and Statistical Analysis

The primary outcome was the proportion of matrix records documenting a validated PROM. Secondary outcomes were any patient-reported endpoint, nonvalidated patient satisfaction, validated observer-rated measurement, mixed patient/observer instruments, any named standardized instrument, exploratory documentation breadth, study designs, reconstructive modalities, anatomical focus, and explicitly recorded limitations. Categorical variables are reported as counts and percentages; study size is summarized by median and interquartile range. Fisher exact tests and unadjusted odds ratios with 95% confidence intervals compared prospective with nonprospective studies. Tests were two-sided, exploratory, and unadjusted for multiplicity or confounding.

Sensitivity analyses were descriptive within BCC-only and mixed-histology strata, by publication period (2015–2020 vs. 2021–2026), after exclusion of the database record, and at documentation-breadth thresholds of at least three, four, or all five domains. The BCC-only stratum is the principal disease-specific analysis; mixed-histology/NMSC studies are complementary and do not provide BCC-specific outcome estimates unless explicitly separated. Multivariable modeling was not attempted because only four records documented a validated PROM. A specialty-stratified analysis was not performed because clinical specialty and author department were not consistently available in the matrix; the journal title was not used as an unreliable proxy. No clinical effect sizes were pooled.

### 2.6. Methodological Characteristics, Bias, and Ethics

No formal risk-of-bias instrument was applied. The narrative matrix lacked the item-level full-text information required for valid design-specific appraisal, and reporting checklists were not repurposed as quality scores. We therefore describe only study architecture and limitations recorded in the matrix. The study used aggregate published information, involved no patient-level data, and required no ethics approval.

## 3. Results

### 3.1. Evidence Corpus and Study Architecture

**Figure 1:**
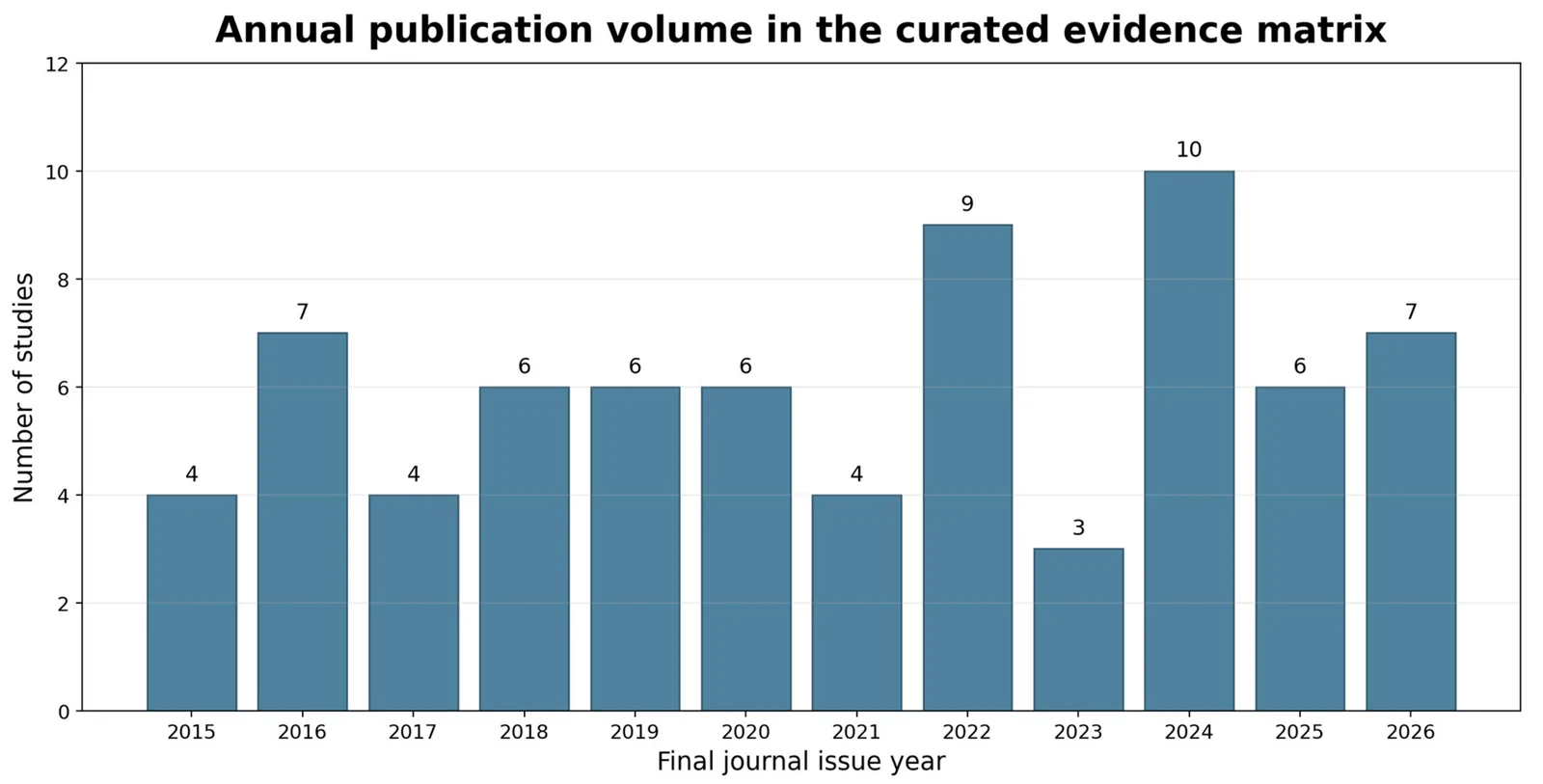
Annual publication volume in the curated 72-record evidence matrix, standardized to final journal issue year. Seven records have 2026 issue dates; the analytic cutoff was 31 July 2026.

The curated analytic corpus comprised 72 records with final journal issue years from 2015 through 2026 and an analytic cutoff of 31 July 2026. In total, 39 records (54.2%) had issue years from 2021 onward, with the largest annual volume in 2024 (10 records) (Figure 1). The United States (*n* = 9), Turkey (*n* = 8), and China (*n* = 7) were the most frequently represented countries. These distributions describe the curated corpus only (Table 1).

Sample size was available for 70 records. The median was 55.5 reported analytic units (IQR 30–148). Analytic units comprised patients, lesions, tumors, cases, or excisions, and were not summed for substantive interpretation. One database record contained 96,458 patients and is identified separately because of its scale [50].

Retrospective designs accounted for 59 records (81.9%); 9 (12.5%) were prospective, 7 (9.7%) comparative, and 3 (4.2%) multicenter. Thirty records (41.7%) were described as case series. No randomized reconstructive trial was represented. In total, 40 records (55.6%) were coded BCC-only, whereas 32 (44.4%) included other skin malignancies or broader NMSC populations. The anatomical focus was general face/head-and-neck in 28, periocular/eyelid in 22, nasal in 17, and other or unclear in 5. BCC-only findings are treated as the disease-specific stratum; mixed studies provide complementary evidence only.

The matrix did not support defensible frequency estimates for detailed diagnostic variables such as primary versus recurrent disease, aggressive histologic features, preoperative biopsy, imaging, or histopathologic margin status. We therefore do not present diagnostic reporting as an analyzed outcome.

### 3.2. Reconstructive Modalities

Local or locoregional flaps were documented in 48 matrix records (66.7%). They appeared in 16 of 17 nasal-focused records (94.1%), 13 of 22 periocular/eyelid records (59.1%), and 19 of 28 general facial/head-and-neck records (67.9%).

**Figure 2:**
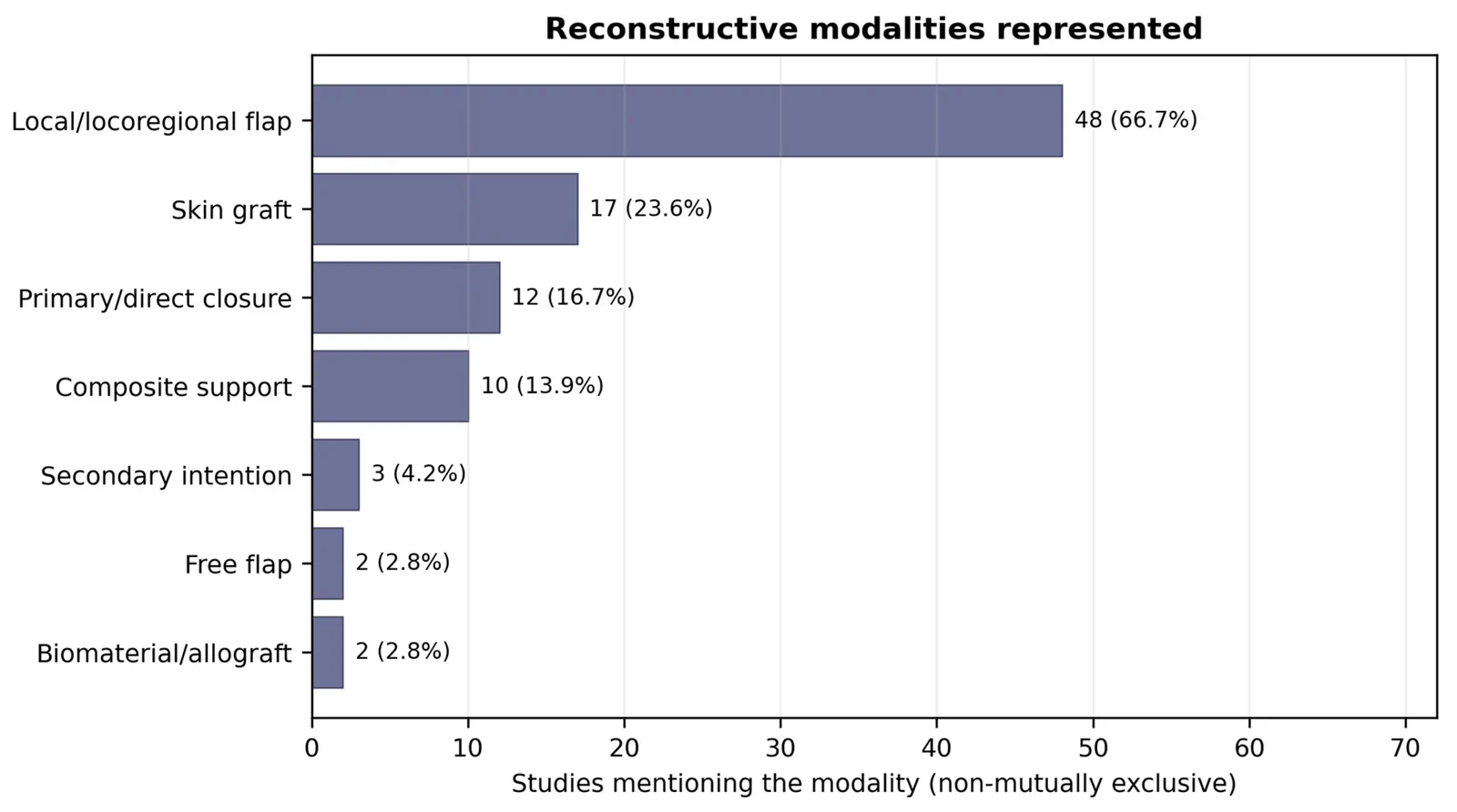
Reconstructive modalities documented in the curated matrix. Categories are non-mutually exclusive.

Skin grafting was documented in 17 records (23.6%), primary or direct closure in 12 (16.7%), and composite lamellar, cartilage, or mucosal support in 10 (13.9%). Secondary intention healing appeared in three records, free-flap reconstruction in two, and biomaterial or allograft strategies in two. Because many records summarized algorithms or multiple procedures, modality categories were not mutually exclusive (Figure 2).

### 3.3. Outcome Domain Reporting

Aesthetic or cosmetic outcomes were documented in 47 matrix records (65.3%), complications or flap viability in 45 (62.5%), oncologic outcomes in 33 (45.8%), functional outcomes in 27 (37.5%), and margin or tissue-sparing outcomes in 15 (20.8%). Nine records (12.5%) documented an endpoint obtained from patients, including validated PROMs and nonvalidated satisfaction assessments.

**Figure 3:**
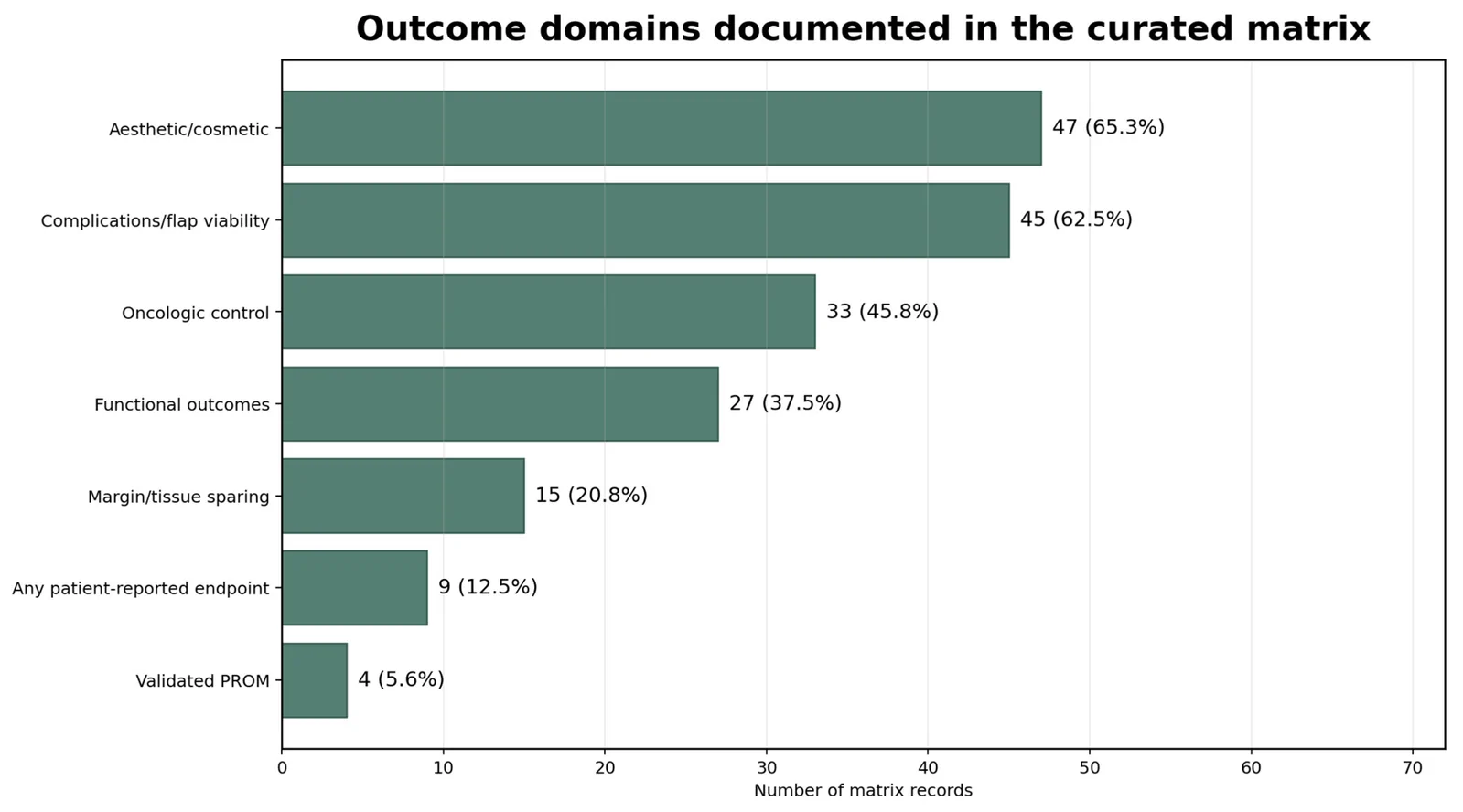
Frequency of outcome domains documented in the curated matrix. A PRO is distinguished from the PROM used to measure it.

Four records (5.6%) documented a validated PROM: two used the patient component of POSAS, one used the Skin Cancer Index together with FACE-Q, and one used the FACE-Q Cancer Worry scale. Five records (6.9%) named any standardized instrument because this broader count additionally included one observer-only VSS assessment. Two POSAS records used mixed patient/observer instruments, one VSS record used a validated observer-only scale, and five patient-reported records used nonvalidated satisfaction assessment. Eight records (11.1%) documented at least four of five domains; the median documentation breadth was two domains (IQR 2–3). No matrix record documented all five domains (Figure 3).

### 3.4. Prospective Design and Patient-Centered Measurement

**Figure 4:**
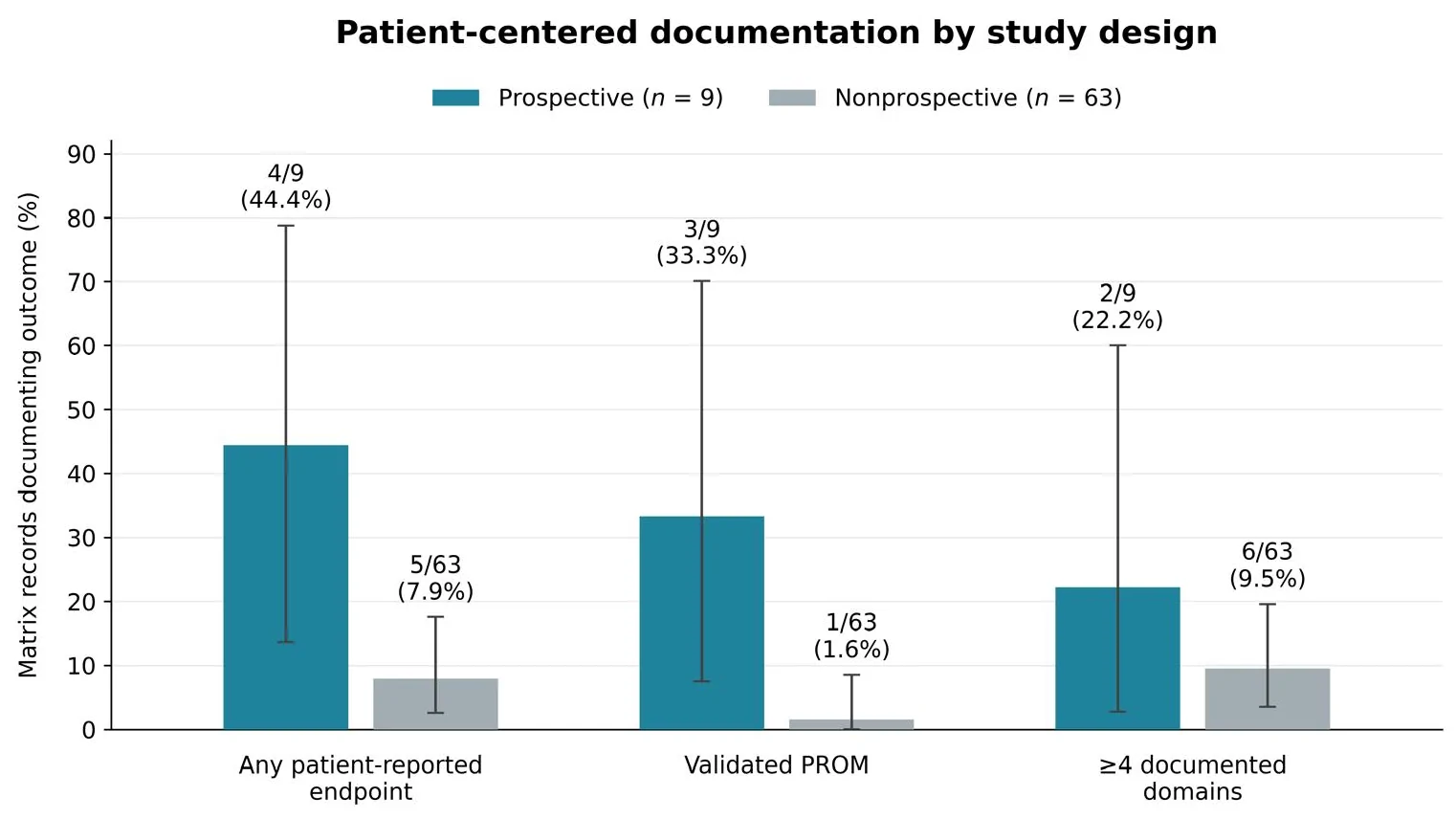
Patient-centered documentation by study design. Labels show raw numerators/denominators and percentages; error bars are exact 95% binomial confidence intervals.

In total, 4 of 9 prospective records (44.4%) documented a patient-reported endpoint, compared with 5 of 63 nonprospective records (7.9%; OR 9.28, 95% CI 1.87–46.01; Fisher exact *p* = 0.011). Three prospective records (33.3%) documented a validated PROM compared with one nonprospective record (1.6%; OR 31.00, 95% CI 2.77–346.32; *p* = 0.005). Any named standardized instrument appeared in three prospective and two nonprospective records (OR 15.25, 95% CI 2.11–110.01; *p* = 0.012). The documentation of at least four domains was numerically more common prospectively (22.2% vs. 9.5%; OR 2.71, 95% CI 0.46–16.13; *p* = 0.260). These unadjusted associations are exploratory and cannot establish that a prospective design yielded better documentation (Table 2; Figure 4).

### 3.5. Disease-Specific and Other Sensitivity Analyses

In the principal disease-specific stratum, 4 of 40 BCC-only records (10.0%) documented a patient-reported endpoint, 1 (2.5%) documented a validated PROM, and 2 (5.0%) named any standardized instrument; only 2 (5.0%) documented at least four domains. The complementary mixed-histology/NMSC stratum contained 5 of 32 patient-reported records (15.6%), 3 validated PROM records (9.4%), and 6 records documenting at least four domains (18.8%). Outcomes from mixed cohorts were not interpreted as BCC-specific estimates.

The validated PROM documentation numbered 1 of 33 records (3.0%) in 2015–2020 and 3 of 39 (7.7%) in 2021–2026; any named standardized instrument increased from 1 of 33 (3.0%) to 4 of 39 (10.3%). Patient-reported endpoint documentation was similar across eras (12.1% vs. 12.8%). Thirty-one records (43.1%) documented at least three of five domains, eight (11.1%) at least four, and none all five. Excluding the database record produced the expected negligible change in unweighted study-level estimates, and is retained only as a denominator-influence check (Table 3; Supplementary Tables S3 and S4).

### 3.6. Recorded Methodological Limitations

A single-center design was recorded for 49 matrix records (68.1%), and an absent comparator or explicitly nonrandomized design for 41 (56.9%). A mixed histology or lack of BCC-specific outcomes was recorded for 21 (29.2%). Nonvalidated or subjective aesthetic assessment was explicitly recorded in 17 summaries, a small sample size in 13, and short follow-up in 7. These are minimum frequencies from narrative summaries, not full-text risk-of-bias judgments (Supplementary Figure S1).

## 4. Discussion

### 4.1. Principal Findings

Within this curated corpus, technical variety contrasted with limited study design breadth and sparse standardized patient-centered measurement. Local and locoregional flaps were commonly documented, whereas more than four in five records were retrospective, fewer than one in ten were comparative, only three were multicenter, and only four documented a validated PROM. These results characterize the supplied matrix; they neither establish the completeness of the literature nor compare reconstructive effectiveness.

We removed ‘Diagnostic Reporting’ from the title because the matrix did not allow the reliable, item-level quantification of tumor size, precise subunit, recurrence status, aggressive histology, biopsy, imaging, margin-control method, final margin status, defect dimensions, or surveillance. These variables remain clinically important and should be captured in future full-text systematic work and prospective registries [1,2].

The median record size of 55.5 analytic units is more informative than a pooled count because patients, lesions, tumors, cases, and excisions were combined across summaries. One 96,458-patient database record [50] would dominate any sum, but had limited procedural detail. We therefore removed the emphasis on the aggregate denominator and treated the exclusion of that record only as a descriptive check.

### 4.2. Reconstructive Modality and Confounding by Indication

The predominance of local flaps is anatomically plausible. Adjacent tissue frequently provides the closest match in color, thickness, texture, adnexal quality, and contour, while allowing scar placement along aesthetic-unit boundaries. In the large Japanese BCC cohort, local flaps were the most common reconstruction, while tumor size and aggressive histology influenced the need for grafting [26]. A comparative Chinese study reported shorter healing, fewer adverse events, lower scar scores, and higher satisfaction with fine suturing and local flaps than with skin grafting in selected patients [40].

Frequency of use must not be interpreted as universal superiority. Defect size and depth, free-margin involvement, cartilage or mucosal loss, comorbidity, anticoagulation, expected operative time, and surgeon experience all influence procedure selection. Prospective cohort evidence in older adults likewise shows that patient characteristics and treatment burden are integral to outcome interpretation [35]. These factors create strong confounding by indication. A graft may be selected for a medically frail patient or for the surveillance of a high-risk bed, while a flap may be selected where contour and functional preservation are paramount. Comparative studies must therefore standardize baseline defect anatomy and model treatment selection rather than compare unadjusted technique groups.

### 4.3. Oncologic Clearance and Reconstruction Form One Pathway

Reconstruction begins with the margin-control strategy. Mohs surgery can preserve substantial healthy tissue in selected infiltrative facial BCC [3,4], and primary tumors generally require fewer stages and leave smaller defects than recurrent or incompletely excised lesions [5]. Margin control, defect measurement, reconstruction, and follow-up should therefore be reported as a single clinical pathway. In the present corpus, fewer than half of studies included an oncologic outcome and only one fifth reported margin, Mohs-stage, defect size, or tissue-sparing measures. A visually successful reconstruction without margin status or recurrence surveillance provides an incomplete account of treatment success. Long-term surveillance is essential; in a randomized comparison of surgical excision and Mohs surgery, a substantial proportion of recurrences were detected after five years during 10-year follow-up [86].

Clinical examination, histopathologic diagnosis, margin assessment, and risk stratification influence the reconstructive pathway [1,2]. In this revision, these variables are presented as clinical context and future reporting priorities, not as quantitatively analyzed diagnostic outcomes.

### 4.4. The Patient Perspective Remains Undermeasured

The matrix documented a patient-reported endpoint in nine records, but these endpoints were methodologically heterogeneous. Four used validated PROMs; five used nonvalidated satisfaction assessment. POSAS combines patient and observer ratings [7], VSS is an observer-rated scar scale [8], and the Skin Cancer Index and FACE-Q are PROMs [9,10,11,12,13]. Surgeon-rated appearance, observer-rated scar quality, and patient satisfaction are therefore reported separately rather than collapsed as equivalent constructs.

Prospective design was associated with more frequent patient-reported and validated-PROM documentation, but only nine records were prospective and only four documented a validated PROM. The resulting confidence intervals were very wide, analyses were unadjusted, and prospective status may be confounded by publication era, journal, study purpose, or specialty. The findings are exploratory descriptive associations, not evidence that prospective design itself improves reporting. The independent systematic review of PROMs after Mohs reconstruction likewise found inconsistent instrument selection [14].

### 4.5. From Additional Case Series to Comparable Research

The corpus suggests priorities for future research rather than comparative effectiveness conclusions. Studies should define defect anatomy, explain treatment selection, report oncologic and functional outcomes at prespecified intervals, and capture patients’ experience with validated PROMs. Prospective registries and multicenter comparisons may be feasible, but a formal research agenda requires full-text evidence synthesis and stakeholder input.

Future work should standardize clinicopathologic context and reconstructive outcomes together. This is a recommendation for subsequent consensus development, not a result directly estimated from the present summary-level corpus.

### 4.6. Author-Proposed Pragmatic Reporting Agenda

Table 4 presents an author-proposed pragmatic reporting agenda prompted by gaps documented in the matrix. It is not a core outcome set and was not derived through stakeholder consensus. Formal development should distinguish what outcomes to measure from how to measure them, and should follow COMET, COS-STAD, and COSMIN methodology [87,88,89]; procedural reporting may draw on SUPER [90]. The proposed follow-up points are pragmatic candidates for future consensus work unless specifically supported by cited evidence.

### 4.7. Strengths and Limitations

Strengths include the transparent restriction of inference to a named curated corpus, the explicit separation of BCC-only and mixed-histology evidence, a clarified PRO/PROM taxonomy, the correction of the main bibliography, and record-level reconciliation across the matrix and references. Supplementary Table S1 contains the complete narrative matrix, Table S2 the revised codebook, and Table S6 the reference and eligibility crosswalk.

The limitations determine interpretation. First, the corpus was not generated by a reproducible search; databases, complete strategies, deduplication, language restrictions, screening procedures, and exclusion reasons are unavailable, and completeness cannot be assessed. Second, coding used narrative summaries rather than duplicate full-text re-extraction, so apparent absence may represent under-ascertainment. Third, one investigator coded the matrix and inter-rater agreement is unavailable. Fourth, patients, lesions, tumors, cases, and excisions cannot be pooled. Fifth, no design-specific risk-of-bias assessment was possible. Sixth, only nine records were prospective and four documented a validated PROM; all odds ratios are sparse, unadjusted, and potentially confounded. Seventh, specialty could not be analyzed reliably because it was not consistently encoded. Finally, 2026 is a partial issue year through 31 July 2026.

Accordingly, this is hypothesis-generating meta-research about documentation within a curated corpus. It does not estimate comparative effectiveness, establish prevalence across the complete literature, or replace a systematic review with duplicate full-text appraisal.

The audit nevertheless identifies internally reproducible patterns within the supplied matrix and provides a corrected, transparent basis for designing a future systematic review and consensus process.

## 5. Conclusions

Within this curated corpus, retrospective and noncomparative designs predominated, local or locoregional flaps were frequently documented, and validated PROM use was uncommon. The BCC-only stratum provided the principal disease-specific description, while mixed-histology cohorts were treated as complementary. Exploratory associations with prospective design were imprecise and noncausal.

A reproducible systematic review with duplicate full-text extraction is needed before field-wide frequency claims can be made. Future prospective studies should integrate clinicopathologic context, defect anatomy, reconstructive rationale, functional and aesthetic assessment, oncologic follow-up, and validated patient-reported measurement.

## Figures and Tables

**Table 1:** Characteristics of the curated evidence corpus.

Characteristic	*n*	% or Summary
Publication period	72	2015–31 July 2026
Sample size available	70	97.2%
Median study size	-	55.5 (IQR 30–148)
Retrospective design	59	81.9%
Prospective design	9	12.5%
Comparative design	7	9.7%
Multicenter design	3	4.2%
Case series	30	41.7%
BCC-only cohort	40	55.6%
Mixed histology/NMSC	32	44.4%
General face/head-and-neck	28	38.9%
Periocular/eyelid	22	30.6%
Nasal	17	23.6%
Other or unclear anatomical focus	5	6.9%

Note: Values are *n* (%) unless otherwise stated. Anatomical focus categories are mutually exclusive. Percentages describe the curated corpus and should not be generalized to the complete literature.

**Table 2:** Exploratory association between prospective design and patient-centered documentation.

Outcome	Prospective	Nonprospective	OR	95% CI	*p*
Any patient-reported endpoint	4/9 (44.4%)	5/63 (7.9%)	9.28	1.87–46.01	0.011
Validated PROM	3/9 (33.3%)	1/63 (1.6%)	31.00	2.77–346.32	0.005
Any named standardized measure	3/9 (33.3%)	2/63 (3.2%)	15.25	2.11–110.01	0.012
≥4 documented domains	2/9 (22.2%)	6/63 (9.5%)	2.71	0.46–16.13	0.260

Note: Values are matrix records with exact numerators/denominators. Odds ratios are unadjusted. CI, confidence interval, patient-reported outcome; PROM, patient-reported outcome measure.

**Table 3:** Descriptive sensitivity analyses of outcome documentation.

Analysis (*n*)	Any Patient-Reported	Validated PROM	Named Standardized	≥4 Domains
Overall curated corpus (*n* = 72)	9 (12.5%)	4 (5.6%)	5 (6.9%)	8 (11.1%)
Excluding database record (*n* = 71)	9 (12.7%)	4 (5.6%)	5 (7.0%)	8 (11.3%)
BCC-only stratum (*n* = 40)	4 (10.0%)	1 (2.5%)	2 (5.0%)	2 (5.0%)
Mixed-histology/NMSC (*n* = 32)	5 (15.6%)	3 (9.4%)	3 (9.4%)	6 (18.8%)
Issue years 2015–2020 (*n* = 33)	4 (12.1%)	1 (3.0%)	1 (3.0%)	4 (12.1%)
Issue years 2021–2026 (*n* = 39)	5 (12.8%)	3 (7.7%)	4 (10.3%)	4 (10.3%)

Note: Percentages use the analysis-specific denominator. The database record analysis is a denominator-influence check; all study-level analyses are unweighted. Mixed histology results are complementary and are not BCC-specific.

**Table 4:** Author-proposed pragmatic reporting agenda for future studies; not a consensus-derived core outcome set.

Domain	Candidate Variables	Pragmatic Candidate Timing	Candidate Measurement Approach
Oncologic safety	Histology; primary/recurrent status; margin-control method; final margin status; re-excision; recurrence	Baseline; surgery; ≥12 months; consider 3–5 years [86]	Pathology-defined variables and time-to-event reporting
Defect anatomy	Subunit; dimensions/area; depth; free margin, cartilage, mucosa, lamella, nerve or bone involvement	After definitive clearance	Standardized measurements and photography
Reconstructive exposure	Closure/graft/flap taxonomy; stages; donor site; structural support; surgeon specialty	Intraoperative and each stage	Standardized operative taxonomy
Complications	Necrosis, flap loss, infection, dehiscence, hematoma, ectropion, obstruction, revision	30 and 90 days; 1 year	Consensus definitions and severity grading
Function	Eyelid closure, lacrimal drainage, nasal airflow, oral competence, sensation	Baseline; 3, 6, 12 months	Anatomy-specific objective tests plus clinician assessment
Appearance/scar	Contour, color/texture match, distortion, scar quality	3, 6, 12 months	POSAS or other validated scar measure; standardized photographs
Patient perspective	Appearance satisfaction, cancer worry, psychosocial impact, treatment burden	Baseline; 3, 6, 12 months	FACE-Q Skin Cancer Module, Skin Cancer Index, validated burden measure
Resource use	Operative time, visits, admissions, stages, time to completion, costs	Through completion	Prospective resource capture

Note: All time points except the extended recurrence-surveillance horizon are pragmatic author proposals requiring stakeholder consensus. The 3–5-year recurrence horizon is informed by randomized 10-year follow-up evidence showing late recurrences [86].

## Data Availability

The data supporting the findings of this study are included in the article and its Supplementary Materials. The original search and screening records for the parent matrix are unavailable. Further inquiries can be directed to the corresponding authors.

## Supplementary Materials

The following supporting information can be downloaded at: https://www.mdpi.com/article/doi/s1, Table S1. Audited narrative evidence matrix; Table S2. Revised operational coding framework; Table S3. Detailed exploratory analyses; Table S4. Documentation-breadth threshold analysis; Figure S1. Limitations explicitly recorded in the narrative matrix; Table S5. STROBE-oriented reporting map; Table S6. Record-level corpus reconciliation; Table S7. Screening of the reviewer-nominated study; Supplementary References S1–S72.

## Abbreviations

The following abbreviations are used in this manuscript:

BCC	Basal cell carcinoma
CI	Confidence interval
COMET	Core Outcome Measures in Effectiveness Trials
COSMIN	Consensus-based Standards for the selection of health Measurement Instruments
COS-STAD	Core Outcome Set-Standards for Development
FACE-Q	Facial Aesthetic Surgery and Quality-of-Life questionnaire
IQR	Interquartile range
NMSC	Non-melanoma skin cancer
OR	Odds ratio
POSAS	Patient and Observer Scar Assessment Scale
PROM	Patient-reported outcome measure
STROBE	Strengthening the Reporting of Observational Studies in Epidemiology
SUPER	Surgical technique reporting guideline
SWiM	Synthesis Without Meta-analysis
VSS	Vancouver Scar Scale
PRO	Patient-reported outcome
