# Supplementary material for: Study Design, Reconstructive Modalities, and Patient-Reported Outcome Documentation in a Curated Corpus of Facial Basal Cell Carcinoma Reconstruction Studies: An Exploratory Meta-Research Analysis
Source: Diagnostics (Basel). 2026 Sep 12;16(18):2952. doi: 10.3390/diagnostics16182952 (PMC13605029; doi:10.3390/diagnostics16182952)
Supplement: Supplementary file 1 [file diagnostics-16-02952-s001.zip › diagnostics-4511005-supplementary.pdf]

Supplementary Material

Study Design, Reconstructive Modalities, and Patient-Reported Outcome Documentation in a Curated Corpus of Facial Basal Cell Carcinoma Reconstruction Studies

Iris-Iuliana Adam, Ruxandra-Cristina Marin, Mihnea Costescu, Liliana Vecerzan, Ioana Roşca, Bogdan Moldovan, Oana Andreia Coman, Raluca-Gabriela Miulescu, Ana-Maria Popa, Alexandru-Petru Ciucu, and Alina Ormenişan

Supplementary data-integrity note

The quantitative analysis used the 72 rows reproduced in Supplementary Table S1. A record-by-record audit confirmed that six publications unrelated to facial skin cancer or reconstruction had been inserted only into the main manuscript bibliography; none appeared in Table S1, Supplementary References S1–S72, or the coded matrix, and none contributed to any result. Removing those six bibliography-only entries restored the intended numbering in the main text: the 72 corpus reports map exactly to references [3–6,18–85]. Supplementary Table S6 provides the row/S-reference/main reference crosswalk. The corpus remains a frozen, non-systematically assembled matrix with an analytic cutoff of 31 July 2026; the apparent absence of a domain means only that it was not documented in the supplied narrative summary.

Table S1. Audited narrative evidence matrix

No.	Author, year	Country	Design/sample	Tumor/site	Reconstruction	Predictors	Outcomes	Key results	Limitations
1	Thakurani et al., 2022 [S1]	India	Retrospective study (n = 68)	Facial basal cell carcinoma	Facial defects reconstructed mainly with V-Y advancement flap, forehead flap and Limberg flap	Age, sex, occupation, sun exposure, Fitzpatrick skin type, lesion location	Flap survival, postoperative complications, recurrence, reconstructive technique selection	No flap failure, infection or wound dehiscence; no recurrence at 1-year follow-up. Local flaps achieved excellent reconstructive outcomes and an anatomical site-based reconstruction algorithm was proposed.	Retrospective, single-center study, small sample size, short follow-up (1 year).
2	Mishra et al., 2024 [S2]	India	Retrospective study (n = 35)	Primary facial BCC; nose most common; forehead, periorbital, cheek, lip and periauricular regions	FTSG for small superficial defects; locoregional flaps for larger or multi-subunit defects	Age, sex, tumor location, number of facial units/subunits involved, flap type	Flap viability, postoperative complications, recurrence, aesthetic outcome	No flap necrosis, wound dehiscence, hematoma or seroma; no recurrence during 6–18 months follow-up. Locoregional flaps provided excellent aesthetic reconstruction for multi-subunit defects following the facial subunit principle.	Retrospective, single-center study, small sample size, short follow-up.
3	Dhar et al., 2026 [S3]	Bangladesh	Retrospective study (n = 62)	Histologically confirmed facial BCC	STSG, FTSG, local advancement flaps	Age, sex, lesion side, wound size, reconstruction type, one- vs two-stage surgery	Complications, re-excision, length of stay, follow-up	STSG was the most frequently used reconstruction; low complication (6.5%) and re-excision (4.8%) rates; surgical excision with appropriate reconstructive planning achieved favorable outcomes.	Retrospective, single-center study, short follow-up (mean 9.7 weeks).
4	Maimaiti et al., 2016 [S4]	China	Retrospective study (n = 17)	Giant BCC of the head and neck (forehead, eyelid, lip, nasolabial region)	Single-stage reconstruction, predominantly local advancement flaps	Tumor size (5–11 cm), anatomical location, reconstruction type	Flap survival, reconstructive success	All patients underwent successful single-stage reconstruction; no postoperative flap failure; local advancement flaps were effective for giant facial BCC.	Retrospective, single-center study, small sample size.

No.	Author, year	Country	Design/sample	Tumor/site	Reconstruction	Predictors	Outcomes	Key results	Limitations
5	Tahir et al., 2024 [S5]	Pakistan	Prospective interventional study (n = 36)	Facial basal cell carcinoma (10–35 mm)	Reconstruction performed after intraoperative frozen-section confirmation of negative margins	Tumor size, facial location, reconstruction technique, frozen-section margin assessment	Margin clearance, reconstructive planning, association between tumor characteristics and reconstruction	Tumor-free margins were achieved in 77.8% of cases at frozen section. Intraoperative frozen section was effective for guiding reconstruction, particularly for lesions requiring complex reconstruction.	Single-center study, small sample size, limited follow-up.
6	Zhang et al., 2020 [S6]	China	Clinical study (n = 86)	Facial basal cell carcinoma	Direct closure, local flap, or split-thickness skin graft after Mohs surgery; adjuvant topical PDT	Reconstruction method, Mohs surgery, adjuvant PDT	Recurrence, clinical efficacy, tissue preservation	No recurrence at 1 year; one recurrence at 2 years. Mohs surgery combined with PDT achieved excellent tumor control while preserving facial appearance.	Single-center study; no comparison group; reconstruction outcomes not analyzed separately.
7	Bejinariu et al., 2020 [S7]	Romania	Case series	Lower eyelid basal cell carcinoma	Lower eyelid reconstruction following tumor excision	Tumor stage, defect location, reconstructive technique	Functional recovery, aesthetic outcome	Favorable local evolution with good functional recovery. Early diagnosis and surgical treatment were associated with better functional and aesthetic outcomes, whereas delayed surgery and recurrent tumors resulted in inferior outcomes.	Small case series, single-center study, sample size not clearly reported.
8	Zgolli et al., 2023 [S8]	Tunisia	Retrospective study (n = 123)	Primary malignant eyelid tumors	Eyelid reconstruction involving anterior/posterior lamella and canthal reconstruction	Tumor location, surgical margins, reconstructive technique	Functional outcome, anatomical outcome, recurrence	Surgical reconstruction achieved favorable functional and anatomical outcomes; recurrence rate was 6.1%.	Mixed tumor types (not exclusively BCC/cSCC); retrospective single-center study.
9	Nishimura et al., 2026 [S9]	Japan	Retrospective study (n = 1149 facial BCCs)	Facial basal cell carcinoma	Primary closure, local flap, skin graft, secondary intention healing	Age, sex, tumor size, anatomical location, histopathological subtype	Determinants of reconstructive method	Local flap was the most common reconstruction. Larger tumors and aggressive histopathological subtypes were independent predictors of skin graft reconstruction, whereas age, sex, and tumor location were not independently associated after multivariate analysis.	Retrospective, single-center study.
10	McCabe et al., 2024 [S10]	Ireland	Retrospective study (n = 88 excisions, 86 patients)	Periocular basal cell carcinoma	Same-day reconstruction following frozen section-controlled excision	Tumor location, histological subtype, frozen-section margin status	Margin clearance, recurrence, reconstructive feasibility	Negative frozen-section margins were achieved in 93% of cases. Overall recurrence rate was 1.4% after a minimum of 3 years. Same-day reconstruction was feasible with excellent oncologic control.	Retrospective, single surgeon, single-center study.
11	Ikoma et al., 2025 [S11]	Japan	Retrospective study (n = 11)	Lower eyelid basal cell carcinoma	Free tarsoconjunctival graft (FTG) for posterior lamellar reconstruction	Defect size, graft dimensions, reconstructive technique	Complications, recurrence, functional and aesthetic outcomes	FTGs provided reliable reconstruction with no major complications and no tumor recurrence during a mean follow-up of 19.3 months. Minor complications were infrequent and asymptomatic. FTGs were suitable for small to moderately sized defects.	Small sample size, retrospective single-center study, selective use of FTGs.

No.	Author, year	Country	Design/sample	Tumor/site	Reconstruction	Predictors	Outcomes	Key results	Limitations
12	Pupić-Bakrač et al., 2025 [S12]	Croatia	Retrospective study (32 GBCCs among 5926 maxillofacial BCCs)	Giant basal cell carcinoma (≥5 cm) of the maxillofacial region	Skin grafts, local flaps, locoregional flaps, and free flaps following extensive ablative surgery	Age, sex, recurrent vs primary tumor, tumor size, local invasion, reconstructive method	Survival, disease control, cosmetic outcome, reconstructive success	Local flaps were the most frequently used reconstructive option (46.9%). Single-stage radical resection with immediate reconstruction achieved excellent oncologic control (5-year DSS 96.9%) with acceptable cosmetic outcomes and low complication rates.	Retrospective, single-center study; limited to giant BCC.
13	Wu et al., 2018 [S13]	China	Retrospective case series (n = 4)	Huge midfacial basal cell carcinoma involving the eyelid and nose	Frontal axial pattern flap combined with hard palate mucosal graft	Extensive midfacial defects requiring composite reconstruction	Functional outcomes, aesthetic satisfaction, tumor recurrence	All patients achieved good eyelid function, satisfactory aesthetic outcomes, and no tumor recurrence during 12–36 months of follow-up.	Very small sample size, single-center study, no comparison group.
14	Ofaiche et al., 2016 [S14]	France	Prospective observational cohort (111 patients, 135 facial BCCs)	Facial basal cell carcinoma	Surgical excision with reconstruction according to defect characteristics	Age-related skin changes, tumor location (H-zone), infiltrative/sclerosing histology	Patient- and observer-rated scar quality (POSAS), aesthetic, functional, and morphological outcomes	Overall patient satisfaction was high. Better outcomes were associated with signs of skin ageing, whereas infiltrative/sclerosing histology and H-zone location were associated with poorer aesthetic and functional results.	Single-center observational study; reconstructive techniques were heterogeneous and not directly compared.
15	Ceder et al., 2021 [S15]	Sweden	Retrospective cohort (903 facial high-risk BCCs in 813 patients)	Primary, recurrent, and incompletely excised facial high-risk basal cell carcinoma	Reconstruction after Mohs micrographic surgery (primary closure and other techniques according to defect)	Primary vs. recurrent/incompletely excised tumor status, Mohs stages, final defect size	Number of Mohs stages, defect size, reconstructive technique	Primary BCCs required fewer Mohs stages and resulted in significantly smaller final defects than recurrent or incompletely excised tumors. Primary tumors were more frequently amenable to primary closure, whereas recurrent/incompletely excised lesions required more complex reconstruction.	Retrospective single-center study; long-term aesthetic and functional outcomes were not assessed.
16	Dölen et al., 2018 [S16]	Turkey	Retrospective case series (n = 22)	Mid-facial skin tumors (13 BCC, 6 SCC, 3 other skin tumors)	Free-style facial artery and lateral nasal artery perforator flaps for moderate-sized mid-facial defects	Defect size (2 × 2 to 5 × 5 cm), facial subunit reconstruction, perforator flap technique	Flap survival, complications, functional and aesthetic outcomes	Free-style facial perforator flaps achieved reliable single-stage reconstruction with excellent functional and aesthetic outcomes. Only one case of transient venous congestion occurred, with no flap loss.	Mixed tumor population; retrospective design; outcomes not reported separately for BCC patients.
17	Derebaşınlıoğlu & Özkaya, 2021 [S17]	Turkey	Retrospective observational study	Nasal basal cell carcinoma and squamous cell carcinoma	Surgical excision with reconstruction according to nasal subunit	Nasal aesthetic subunit, tumor type, age, sex	Association between tumor location and reconstruction method	Tumor histology (BCC vs. SCC) was not associated with nasal subunit distribution. The nasal aesthetic subunit was the only factor significantly associated with the reconstructive technique used.	Mixed BCC/SCC cohort; sample size not reported in the abstract; outcomes not analyzed separately for BCC.

No.	Author, year	Country	Design/sample	Tumor/site	Reconstruction	Predictors	Outcomes	Key results	Limitations
18	Unlu et al., 2024 [S18]	Turkey	Comparative retrospective study (43 patients)	Eyelid basal cell carcinoma	Reconstruction following one-step surgery (OSS) versus two-step surgery (TSS)	Tumor size, safety margin width, surgical strategy, tumor location	Margin status, reconstructive procedures, lacrimal system preservation, recurrence	Larger tumors were treated with TSS using smaller safety margins. OSS used wider margins but had a higher rate of positive histological margins (not statistically significant). The overall number of reconstructive procedures was similar between groups, while TSS enabled significantly better preservation and reconstruction of the nasolacrimal drainage system.	Small sample size; single-center retrospective study; limited long-term functional and aesthetic outcome assessment.
19	Aristokleous et al., 2022 [S19]	Sweden	Retrospective cohort (143 facial BCCs in 138 patients)	Aggressive and recurrent facial basal cell carcinoma	Reconstruction following Mohs surgery through a multidisciplinary approach involving plastic surgeons	Primary vs. recurrent tumors, multidisciplinary management	Recurrence, interdisciplinary management, reconstructive pathway	Mohs surgery achieved a low recurrence rate (4.9% after a median follow-up of 24 months). A multidisciplinary approach integrating dermatologic surgery, pathology, and plastic reconstruction was feasible and effective for managing complex facial BCCs.	Retrospective design; no comparison of reconstructive techniques; functional and aesthetic outcomes not reported.
20	van Winden et al., 2022 [S20]	Netherlands	Prospective multicenter cohort (539 patients ≥70 years)	Head and neck basal cell carcinoma	Surgical treatment (Mohs micrographic surgery or conventional excision) with reconstruction as clinically indicated	Tumor diameter, complications, frailty (iADL dependency), polypharmacy, comorbidity, sex	Treatment burden, cosmetic outcome, complications, overall survival	Cosmetic outcomes were generally good. Greater treatment burden was independently associated with iADL dependency, female sex, postoperative complications, larger tumor diameter, and polypharmacy. No significant differences in treatment burden or cosmetic outcomes were found between Mohs surgery and conventional excision after adjustment for confounders.	Observational design with possible selection bias; reconstructive techniques were not analyzed separately.
21	Balchev et al., 2022 [S21]	Bulgaria	Retrospective case series (n = 15)	Medial canthal tumors following excision	V-Y glabellar rotation, advancement, or transposition flap	Defect location (upper, medial, lower medial canthus), glabellar flap technique	Functional outcome, cosmetic outcome, need for revision surgery	All patients achieved satisfactory functional outcomes, most had good cosmetic results, and no secondary reconstructive procedures were required during follow-up.	Small sample size; mixed tumor population; no comparison group; histological diagnoses not reported separately.
22	Tamaș et al., 2018 [S22]	Romania	Retrospective study (116 patients)	Facial malignant tumors (70 BCC, 35 SCC, 11 melanoma); most commonly orbital region and nasolabial sulcus	Reconstruction with local flaps (frontal, glabellar, nasolabial flaps and Z-plasty) according to defect location	Tumor histology, anatomical location, defect characteristics guiding flap selection	Reconstruction strategy, re-excision, oncological follow-up	Local flaps were the most frequently used reconstructive method. Reconstruction was tailored to tumor location and pathology. Fifteen patients required re-excision, and multidisciplinary cooperation between surgeons and pathologists contributed to favorable functional and aesthetic outcomes.	Retrospective single-center study; mixed tumor population; no comparative analysis of reconstructive techniques or standardized cosmetic assessment.

No.	Author, year	Country	Design/sample	Tumor/site	Reconstruction	Predictors	Outcomes	Key results	Limitations
23	van Kester et al., 2019 [S23]	Netherlands	Prospective single-center study (256 primary infiltrative BCCs)	Primary infiltrative basal cell carcinoma	Mohs micrographic surgery; postoperative defect area measured (no reconstruction analyzed)	Infiltrative histological subtype; tumor size; surgical defect size	Tissue-sparing effect of Mohs compared with hypothetical standard excision	Mohs surgery reduced the postoperative defect area by a median of 46.4% compared with a calculated 5 mm margin standard excision, demonstrating substantial preservation of healthy tissue.	Single-center study; no randomized comparison; reconstructive techniques and postoperative functional/aesthetic outcomes were not evaluated.
24	Egeler et al., 2019 [S24]	USA	Retrospective single-center study (418 patients)	Facial defects after Mohs surgery; 73% BCC, 14% SCC; nose most commonly involved (50%)	Reconstruction according to facial aesthetic unit using local advancement flaps, skin grafts, and other techniques	Defect size, facial aesthetic unit, timing of reconstruction (same day vs. delayed), reconstructive modality	Reconstruction type, complications, local recurrence, scar revision	Local advancement flaps were used in 51% and grafts in 25% of reconstructions. Overall complication rate was 3%, local recurrence 4%, and scar revision 6%. Same-day reconstruction was associated with a higher proportion of complications than delayed reconstruction.	Retrospective single-center design; heterogeneous tumor population; no standardized patient-reported aesthetic assessment.
25	Gniadecki et al., 2015 [S25]	Denmark	Retrospective study (231 patients, 263 BCCs)	Facial basal cell carcinoma; forehead (31.3%), nose (31.0%), cheek (14.7%)	Mohs micrographic surgery followed by primary closure or local flap reconstruction	Tumor location, primary vs. recurrent BCC, defect size after Mohs	Defect size, reconstructive method, recurrence, cosmetic and functional outcomes	Mohs surgery produced 40% smaller defects than estimated standard excision margins. Reconstruction was achieved by primary closure in 49% and local flaps in 40% of cases. No recurrences were observed during follow-up, and cosmetic and functional outcomes were reported as excellent.	Retrospective single-center study; relatively short follow-up; cosmetic outcomes were not assessed using validated patient-reported measures.
26	Balchev, 2024 [S26]	Bulgaria	Retrospective cohort (450 eyelid tumors)	Eyelid tumors, including BCC	Eyelid reconstruction following tumor excision using standard oculoplastic techniques	Tumor location, ciliary margin involvement, canthal location, surgical technique	Recurrence, postoperative complications	Recurrence occurred in 2.8% of cases and complications in 7.0%. Recurrences were associated with ciliary margin involvement and tumor location, while the choice of surgical technique influenced both complication and recurrence rates.	Mixed tumor population; retrospective single-center study; reconstructive techniques were not analysed separately according to histology.
27	Chen et al., 2025 [S27]	China	Retrospective comparative study (46 patients; 23 local flap + fine suturing vs. 23 skin graft)	Basal cell carcinoma of the head and face	Extended tumor excision with frozen-section margin control followed by local flap reconstruction with fine suturing or conventional skin grafting	Reconstruction method (local flap vs. skin graft)	Operative time, healing time, adverse events, Vancouver Scar Scale (VSS), patient satisfaction	Local flap reconstruction with fine suturing resulted in shorter operative and healing times, fewer postoperative adverse events, lower VSS scar scores, and higher patient satisfaction compared with skin grafting.	Small single-center retrospective study; non-randomized design; limited sample size.
28	Toman et al., 2022 [S28]	USA	Retrospective propensity score-matched case-control study (286 patients; 143 matched pairs)	Basal cell carcinoma and squamous cell carcinoma of the face, head, and neck treated with Mohs surgery	Mohs defects reconstructed with dehydrated human amnion/chorion membrane (dHACM) versus autologous local flaps or full-thickness skin grafts	Reconstruction method (placental allograft vs. autologous tissue)	Infection, scar cosmesis, scar revision, reoperation, postoperative morbidity	dHACM reconstruction was associated with significantly lower risks of infection, poor scar cosmesis, scar revision, and reoperation compared with flap or full-thickness skin graft reconstruction, with no increase in overall postoperative complications.	Retrospective non-randomized design; single treatment network; potential selection bias despite propensity matching.
29	Mathieu et al., 2024 [S29]	France	Retrospective single-center observational study (426 head and neck skin cancers)	Head and neck skin cancers; BCC (77%) and SCC (18%); nose and cheek most frequent sites	Surgical excision followed predominantly by local flap reconstruction (51%) or primary closure (34%)	Tumor location, histological subtype, reconstructive approach	Surgical margins, recurrence/progression, reconstructive strategy distribution	Local flaps were the most frequently used reconstructive technique (51%). Clear margins were achieved in 65% of cases, and local recurrence/progression	Primarily epidemiological study; no comparison of reconstructive techniques; no functional or aesthetic outcome assessment.

No.	Author, year	Country	Design/sample	Tumor/site	Reconstruction	Predictors	Outcomes	Key results	Limitations
								occurred in only 2% during follow-up.	
30	Mutlu et al., 2016 [S30]	Turkey	Retrospective case series (23 patients)	Periorbital defects following tumor resection	Reconstruction selected according to periorbital aesthetic subunits using primary closure, skin grafts, local flaps, forehead flaps, Mustardé, Tenzel, Tripier, glabellar and bilobed flaps	Defect location, extent of tissue loss, aesthetic subunit involvement	Reconstructive strategy, functional and aesthetic outcomes	Reconstruction tailored to aesthetic subunits achieved functionally and aesthetically acceptable outcomes across a range of periorbital defects, supporting an algorithmic approach to flap selection.	Small single-center retrospective study; mixed defect etiologies; no comparison group; tumor histology not reported separately.
31	Marcasciano et al., 2017 [S31]	Italy	Retrospective cohort (310 patients)	Nasal non-melanoma skin cancers (predominantly BCC, with SCC included)	Post-oncologic nasal reconstruction using locoregional flaps; defects classified by proximal, middle, distal, or complex subunits	Defect location, anatomical subunit involvement, defect complexity	Reconstructive strategy, functional and aesthetic outcomes	Locoregional flaps provided reliable reconstruction across all nasal subunits. Defect location and anatomical subunit involvement guided flap selection, allowing satisfactory functional and aesthetic restoration after tumor excision.	Retrospective single-center study; mixed NMSC population; no comparative analysis between flap techniques; outcomes mainly descriptive.
32	DeWitt and Holmes, 2019 [S32]	USA	Retrospective case series (12 patients)	Preauricular tumors treated with Mohs surgery (9 BCC, 2 SCC, 1 melanoma)	Medium-to-large preauricular defects reconstructed with a flipped island pedicle flap	Defect size and preauricular location requiring local flap reconstruction	Flap success, aesthetic outcome, postoperative complications	The flipped island pedicle flap achieved successful reconstruction in all patients, with good cosmetic outcomes and no reported postoperative complications.	Small single-center case series; no comparison group; includes one melanoma case.
33	van Hensbergen et al., 2022 [S33]	Netherlands	Prospective longitudinal cohort (151 patients; 99 completed 1-year follow-up)	Facial non-melanoma skin cancer (predominantly BCC and SCC)	Surgical treatment of facial NMSC (including Mohs surgery)	Previous facial skin cancer and previous facial surgery	FACE-Q Skin Cancer – Cancer Worry score (PROM)	Cancer worry decreased significantly between baseline and 3 months after surgery and remained stable at 1 year. Patients with previous facial NMSC or prior facial surgery showed less improvement in cancer worry.	Mixed NMSC population; reconstruction techniques not analyzed; psychological rather than reconstructive outcomes.
34	Zlatarova et al., 2016 [S34]	Bulgaria	Retrospective case series (39 patients)	Eyelid carcinomas following surgical excision	Anterior lamellar reconstruction using full-thickness skin grafts; Hughes tarsoconjunctival flap used for large full-thickness lower eyelid defects	Tumor location, tumor size, postoperative defect size, graft donor site	Graft viability, postoperative complications, eyelid function, cosmetic outcome	Full-thickness skin grafts remained viable in 95% of cases. Early complications were infrequent and managed conservatively. All patients achieved normal postoperative eyelid function, and cosmetic outcomes were rated as good (36%) or excellent (64%)	Retrospective single-center study; no comparison group; mixed eyelid carcinoma population; outcomes based on clinical assessment rather than validated PROMs.
35	Romzova et al., 2024 [S35]	Slovakia	Prospective observational study (87 patients)	Lower eyelid malignant tumors: BCC (86%), SCC (8%), melanoma (5%), Merkel cell carcinoma (1%)	Lower eyelid reconstruction using primary closure, local flaps, skin grafts, Hughes flap, Hewes flap, periosteal flap, nasal chondromucosa, conchal cartilage and buccal mucosa according to defect characteristics	Tumor size, histopathological type, tumor location, defect size and lamellar involvement	Reconstructive strategy, defect characteristics, functional and aesthetic restoration	Larger tumors were associated with higher tumor grade and larger excision defects. Tumor location and histology significantly influenced defect size and reconstructive choice. Local flaps were the most common method for anterior lamellar reconstruction, whereas posterior lamellar defects required cartilage, mucosal grafts or flap reconstruction according to defect extent.	Single-center prospective study; no comparison between reconstructive techniques; functional and aesthetic outcomes were not assessed using validated outcome measures.

No.	Author, year	Country	Design/sample	Tumor/site	Reconstruction	Predictors	Outcomes	Key results	Limitations
36	Jung et al., 2024 [S36]	South Korea	Retrospective case series (12 patients)	Temple skin cancers: SCC (6), BCC (5), myxofibrosarcoma (1)	Medium-sized temple defects reconstructed with lateral arm free flaps following wide tumor excision	Temple subunit defects requiring free tissue transfer	Flap survival, postoperative complications, functional outcome, donor-site morbidity	All lateral arm free flaps survived successfully with no major complications. Good functional outcomes and preserved donor-arm range of motion were achieved in all patients, demonstrating the feasibility of this technique for medium-sized temporal defects.	Small single-center case series; no comparison group; includes one non-NMSC case; aesthetic outcomes were not assessed using validated measures.
37	Garg et al., 2025 [S37]	USA	Retrospective database cohort study (TriNetX; 96,458 matched patients, 48,229 immediate vs. 48,229 staged reconstruction)	Facial non-melanoma skin cancer (BCC and SCC) following Mohs micrographic surgery	Immediate (<24 h) versus staged (>24 h) facial reconstruction	Timing of reconstruction	Overall complications, wound dehiscence, flap loss, skin necrosis, infection, hematoma, seroma, hypertrophic scarring, hyperpigmentation, revision surgery	Immediate reconstruction was associated with a lower risk of overall complications at 6 months, significantly lower wound dehiscence at 1 month, and fewer revision procedures at 12 months. Rates of flap loss, skin necrosis, hematoma, hypertrophic scarring, and hyperpigmentation were similar between groups.	Retrospective database study; limited procedural detail regarding reconstructive techniques; potential residual confounding despite matching.
38	Chen et al., 2026 [S38]	China	Prospective observational study (36 patients)	Lower lip basal cell carcinoma and squamous cell carcinoma (T1–T2N0M0)	Full-thickness lower lip defects reconstructed using a subcutaneous tissue pedicle flap combined with a labial mucosa flap	Age, defect size, wound-care method	Functional outcome, aesthetic outcome, wound healing, scar quality, recurrence	The combined flap provided reliable functional restoration and satisfactory cosmetic outcomes. Delayed healing was more frequent in patients >70 years and in defects >2 cm, while larger defects had lower functional scores. Recurrence was 10.5% for SCC and 5.8% for BCC during 6–24 months of follow-up.	Single-center prospective study; no comparison group; limited to lower lip reconstruction.
39	Grieco et al., 2018 [S39]	Italy	Retrospective case series (86 patients)	Nasal skin cancer: BCC (72%), SCC (28%)	Nasal defects reconstructed using the modified Zitelli bilobed flap	Nasal defect reconstruction following tumor excision	Postoperative complications, functional preservation, cosmetic outcome, short- and long-term follow-up	Reconstruction with the modified Zitelli bilobed flap resulted in no infections or major complications. Nasal function and facial contour were well preserved, with only minor alar rim distortion in 12 patients, who nevertheless reported satisfactory cosmetic outcomes.	Single-center case series without a comparison group; aesthetic outcomes were not assessed using validated patient-reported measures.
40	Uzun et al., 2015 [S40]	Turkey	Retrospective case series (163 patients)	Nasal non-melanoma skin cancer: BCC (121), SCC (42)	Immediate reconstruction following tumor excision using primary closure or local flaps (Limberg, Miter, glabellar, bilobed, nasolabial, V-Y advancement, forehead flap)	Nasal defect location and size guiding flap selection	Reconstructive strategy, defect distribution, aesthetic reconstruction	Local flap selection was tailored according to the anatomical location of the nasal defect, and the authors proposed a practical algorithm to optimize aesthetic reconstruction while achieving complete tumor excision.	Single-center retrospective study; no comparison group; limited reporting of complications and no validated functional or aesthetic outcome measures.
41	Kofler et al., 2022 [S41]	Germany	Prospective observational study (37 patients)	Nasal BCC and SCC requiring alar reconstruction	Nasal alar defects reconstructed with local flaps modified using a single-stage all-layer shaping suture	Nasal alar defect requiring contour reconstruction after tumor excision	Aesthetic outcome, functional outcome, flap viability, postoperative complications, patient and physician satisfaction	All flaps healed successfully without persistent vascular compromise. At 4 weeks, aesthetic outcomes were rated as good or very good by 73% of physicians and 78.4% of patients, with excellent functional preservation and sustained results at 6-month follow-up.	Single-center prospective study; small sample size; no comparison group.

No.	Author, year	Country	Design/sample	Tumor/site	Reconstruction	Predictors	Outcomes	Key results	Limitations
42	Galindo-Ferreiro et al., 2023 [S42]	Spain/Brazil	Retrospective case series (10 patients)	Lower eyelid tumors: BCC (8), SCC (1), malignant schwannoma (1)	Large (50–75%) full-thickness lower eyelid defects reconstructed using a lateral eyelid island flap with temporal myocutaneous advancement flap	Large central and medial lower eyelid defects after tumor excision	Flap viability, eyelid position and contour, ectropion, aesthetic satisfaction	All flaps remained viable without ischemia. Only one patient developed mild ectropion, and all patients reported satisfactory aesthetic outcomes, supporting the technique as an effective single-stage reconstruction.	Small single-center case series; no comparison group; includes one non-NMSC tumor; no validated aesthetic outcome measures.
43	Gu et al., 2026 [S43]	China	Clinical case series (24 patients)	Nasal basal cell carcinoma	Nasal defects reconstructed using a nasolabial fold retrograde island flap with immediate local heparin microinjection	Flap size, postoperative venous congestion, local anticoagulation	Flap survival, venous congestion, safety, aesthetic outcome	All flaps survived (100%). Three patients (12.5%) developed early venous congestion, which resolved with continued local heparin treatment. No clinically significant systemic coagulation abnormalities or major adverse events were observed.	Single-center case series without a control group; primarily technique-oriented study; limited long-term validated functional and aesthetic assessment.
44	Kinde et al., 2024 [S44]	USA	Prospective observational study (45 patients)	Periocular non-melanoma skin cancer (BCC/SCC)	Surgical excision with periocular reconstruction	Demographic and clinical factors influencing postoperative quality of life	Skin Cancer Index (SCI), FACE-Q, patient-reported quality of life	Quality of life improved significantly by 3 months after surgery. SCI and FACE-Q scores demonstrated better overall well-being and reduced treatment-related adverse effects. Several demographic and clinical variables were identified as predictors of postoperative QoL.	Reconstruction techniques were not analyzed individually; no comparison between reconstructive methods; primary outcome focused on patient-reported quality of life rather than surgical complications.
45	Bascón Rodríguez et al., 2025 [S45]	Spain	Multicenter retrospective case series (35 patients)	Facial skin tumors, predominantly basal cell carcinoma; most commonly nasal tip, nasal ala, melolabial fold, retroauricular region	Facial defects reconstructed using the puzzle advancement flap	Facial subunit and defect location guiding flap application	Early and late complications, recurrence, clinical outcome	Approximately 90% of patients experienced no immediate or late complications. Trapdoor deformity was the most frequent complication, and no tumor recurrences were reported during follow-up. The puzzle flap proved versatile across several facial aesthetic subunits.	Retrospective design; no comparison group; limited reporting of validated functional and aesthetic outcomes; tumor histology distribution incompletely described.
46	Aggarwal et al., 2018 [S46]	USA	Retrospective consecutive case series (30 patients)	Periocular non-melanoma skin cancer defects following Mohs surgery; lower eyelid	Lower eyelid reconstruction using a modified Hughes tarsoconjunctival flap with amniotic membrane graft	Large post-Mohs lower eyelid defects reconstructed with modified Hughes technique	Postoperative complications, eyelid margin appearance, functional reconstruction	No patients developed a hyperemic or hypertrophic eyelid margin during follow-up. The addition of an amniotic membrane graft resulted in favorable reconstructive outcomes with minimal postoperative complications.	Retrospective, non-comparative design; short follow-up; tumor histology not fully reported; no comparison with the standard Hughes procedure.
47	Neuner et al., 2021 [S47]	USA	Retrospective cohort study (1,500 patients)	Facial basal cell carcinoma and cutaneous squamous cell carcinoma treated with Mohs surgery	Facial defects reconstructed using multiple techniques, predominantly advancement flaps	Patient demographics, defect size and location, reconstruction type, surgeon specialty, complications	Complications, undesirable sequelae, need for ancillary aesthetic procedures	Complications occurred in 15.9% of cases, most commonly hypertrophic scars. Advancement flaps were the most frequently used reconstruction (44.3%). Female sex, lip and nasal defects, and transposition flaps were associated with a greater likelihood of postoperative corticosteroid injections, while younger age, female sex, and dermatologist-performed repairs predicted pulsed dye laser treatment. Awareness of patient and defect characteristics may help optimize reconstructive	Retrospective design; heterogeneous reconstructive techniques; aesthetic outcomes assessed indirectly through ancillary interventions rather than validated PROMs.

No.	Author, year	Country	Design/sample	Tumor/site	Reconstruction	Predictors	Outcomes	Key results	Limitations
								planning.	
48	Abdelmofeed, 2022 [S48]	Egypt	Retrospective case series (30 patients)	Medial canthal tumors; medial canthus	Medial canthal defects reconstructed using a glabellar superciliary bilobed flap	Defect size, depth, and location guided flap modification	Defect closure, cosmetic outcome, complications, reoperations	Complete donor-site closure was achieved in all patients without wound dehiscence. Cosmetic outcomes were highly satisfactory to both surgeons and patients, with no intraoperative complications and minimal postoperative morbidity. The technique proved to be an effective single-stage reconstructive option for medial canthal defects.	Retrospective design; no control group; tumor histology not fully specified; aesthetic outcomes assessed subjectively without validated PROMs.
49	Altin Ekin and Karadeniz Ugurlu, 2019 [S49]	Turkey	Retrospective case series (31 patients)	Medial canthal tumors (74.2% basal cell carcinoma)	Medial canthal defects with or without upper/lower eyelid involvement reconstructed using various local flap techniques	Extent of eyelid involvement (upper, lower, both eyelids) guided reconstructive strategy	Functional outcome, cosmetic outcome, complications	Functional outcomes were normal in 96.8% of patients, cosmetic outcomes were satisfactory in all cases, and complications were infrequent (6.4%), consisting of one flap necrosis and one lid-margin notching. Tailoring reconstruction according to eyelid involvement achieved favorable functional and aesthetic results.	Retrospective design; no comparison group; heterogeneous reconstructive techniques; cosmetic outcomes assessed without validated patient-reported measures.
50	Brandão et al., 2020 [S50]	Brazil	Retrospective cross-sectional study (975 cases)	Facial and extra-facial keratinocyte carcinomas treated with Mohs surgery (predominantly BCC)	Multiple reconstructive techniques following Mohs surgery, including primary closure, flaps and grafts	Histological subtype, anatomical location, number of Mohs stages, defect characteristics	Type of reconstruction selected and factors associated with repair choice	Primary linear closure was the most common repair (39%) and was associated with fewer Mohs stages. Nasal defects and tumors requiring ≥2 Mohs stages were significantly more likely to require flap or graft reconstruction than primary closure.	Retrospective design; surgeon preference may have influenced reconstruction choice; includes both facial and extra-facial tumors.
51	Fabrizio, 2015 [S51]	Italy	Retrospective case series (187 patients)	Facial defects after tumor excision, predominantly middle third of the face	Reconstruction using the retroangular flap	Vascular anatomy, flap vascular variations, complications, facial defect location	Morphological and cosmetic outcomes, flap success, complications, long-term follow-up	Reconstruction was successful in the entire cohort with favorable long-term morphological and cosmetic outcomes. Analysis of vascular variations helped define the indications and limitations of the retroangular flap, supporting it as a reliable first-line option for reconstruction of middle facial defects.	Retrospective design; no comparison group; tumor histology not specified; cosmetic outcomes assessed without validated patient-reported measures.

No.	Author, year	Country	Design/sample	Tumor/site	Reconstruction	Predictors	Outcomes	Key results	Limitations
52	Steele et al., 2021 [S52]	USA	Retrospective case series (24 patients)	Malignant skin defects (BCC, SCC, melanoma and others); facial and extremity sites	Reconstruction using the triangular dart flap following tumor excision	Defect size, anatomical location, comorbidities, smoking history	Postoperative complications and reconstructive outcomes	The triangular dart flap provided successful single-stage reconstruction with no major complications and only minor complications in 20.8% of patients, allowing tension-free closure of selected Mohs defects.	Small retrospective series; mixed tumor types and anatomical sites; no comparison group; limited assessment of aesthetic outcomes.
53	Rickstrew et al., 2024 [S53]	Multicenter	Retrospective multicenter case series (66 cases)	Skin cancers of the distal nose treated with Mohs surgery	Nasal tip rotation flap (NTRF)	Tumor size, defect size, anatomical location, Mohs stages, repair dimensions	Postoperative complications and reconstructive success	NTRF proved to be a reliable option for distal nasal defects with no significant postoperative complications.	Retrospective design; no comparison group; mixed skin cancer histologies; limited long-term aesthetic assessment.
54	La Padula et al., 2017 [S54]	Italy	Retrospective case series (24 patients)	Basal cell carcinoma of the nasal tip	Nasal tip defects reconstructed with a levator alae nasi muscle V-Y island flap	Tumor location, defect size (up to 1.8 cm), flap design	Aesthetic outcome, scar quality, maintenance of nasal tip projection	The flap achieved satisfactory aesthetic results, preserved nasal tip projection, and resulted in well-healed scars following BCC excision.	Retrospective design; no comparison group; subjective aesthetic assessment; limited long-term follow-up.
55	Goldman and Wollina, 2020 [S55]	Brazil	Retrospective case series (52 patients)	Nasal BCC (49) and SCC (3)	Nasal defects reconstructed using nasolabial, bilobed, Rintala, tunneled island and forehead flaps	Tumor location, defect size/depth, reconstructive technique	Complications, recurrence, reconstructive outcomes	Nasolabial flap was the most frequently used reconstruction (76.9%). Complications were uncommon and recurrences were rare during follow-up.	Retrospective design; single-center study; no comparative analysis between reconstructive techniques.
56	Rougier et al., 2019 [S56]	France	Retrospective single-center study (132 patients)	Orbito-palpebral non-melanoma skin cancers (71.9% BCC, 22.9% SCC)	Direct closure, skin grafts, local flaps, and other reconstructive techniques	Tumor size, histology, location, excision margins, reconstruction type, sex	Oncological outcomes, aesthetic outcomes, quality of life, healing	Larger tumors, BCC histology and complex location were associated with higher rates of inadequate margins. Flap-based reconstruction was associated with improved surgical margin outcomes.	Retrospective design; single-center study; mixed tumor histologies; aesthetic assessment partly based on self-report.
57	Altin Ekin and Ugurlu, 2020 [S57]	Turkey	Retrospective study (58 patients)	Lower eyelid defects after tumor excision	Modified Hughes flap with advancement flap or full-thickness skin graft for anterior lamella reconstruction	Defect size, reconstruction type, patient characteristics	Functional outcome, cosmetic outcome, complications, secondary surgery	Functional success reached 96.6% and cosmetic success 94.8%. Complication and reoperation rates did not differ significantly between advancement flaps and skin grafts, and no independent risk factor significantly affected procedural success.	Retrospective design; single-center study; tumor histology not fully detailed in the abstract; no randomized comparison.
58	Lindsay and Morton, 2015 [S58]	United Kingdom	Consecutive case series (21 patients)	Basal cell carcinoma of the nasal ala	Combined subcutaneous fat transposition flap with full-thickness skin graft	Defect size (up to 17 mm), nasal alar location, reconstructive technique	Complications, graft survival, aesthetic contour and symmetry	No hematoma, infection or graft failure occurred. The combined flap-graft technique achieved excellent contour restoration and preservation of the nasofacial sulcus with minimal morbidity.	Single-center, non-comparative case series; small sample size; subjective aesthetic assessment; no long-term comparative outcomes.
59	Inozu et al., 2016 [S59]	Turkey	Retrospective case series (12 patients)	Basal cell carcinoma of the nasal tip and supratip	Nasal tip defects reconstructed with the pincer flap	Defect location, defect size (2–3 cm), flap design	Complications, recurrence, aesthetic outcome	No flap loss or tumor recurrence occurred. Minor infection and venous congestion developed in two patients but resolved without additional surgery. Aesthetically favorable single-stage reconstruction was achieved.	Small retrospective series; no comparison group; single-center experience; subjective aesthetic assessment.
60	Almubarak et al., 2026 [S60]	Canada	Retrospective case series (26 patients)	Periocular BCC (18) and SCC (8) involving full-thickness eyelids and canthi	Paramedian forehead flap with adjunctive posterior lamellar reconstruction as needed	Tumor pathology, defect location (medial/lateral canthus), reconstructive strategy	Flap survival, complications, eyelid function, ocular surface outcomes	No flap losses, infections, or major complications occurred. All patients achieved satisfactory eyelid closure with favorable	Retrospective single-center study; mixed BCC/SCC cohort; no comparison group; limited standardized aesthetic assessment.

No.	Author, year	Country	Design/sample	Tumor/site	Reconstruction	Predictors	Outcomes	Key results	Limitations
								functional outcomes.	
61	Kimura et al., 2026 [S61]	Japan	Retrospective single-center study (129 patients)	Facial skin cancers (103 BCC, 26 SCC)	Secondary intention healing versus surgical closure after tumor excision	Defect size, tumor location (NEET), sex, reconstruction method	Cosmetic outcome, epithelialization time, predictors of favorable healing	Cosmetic outcomes were comparable between SIH and surgical closure. Defects ≤14 mm and concave NEET locations were associated with better cosmetic results, while larger defects and female sex independently predicted less favorable outcomes.	Retrospective design; cosmetic assessment available for a subset of patients; single-center Japanese cohort.
62	Al Harthi et al., 2025 [S62]	South Korea	Retrospective study (152 patients)	Eyelid malignancies (52.6% BCC)	Eyelid reconstruction following tumor excision using techniques tailored to defect characteristics	Tumor type, size, location, extent, reconstructive method	Complications, recurrence, prognostic factors	Direct closure was the most common reconstruction. Complication rates were low. Lymph node involvement and positive frozen margins were independent predictors of local recurrence.	Retrospective single-center study; mixed eyelid malignancies; reconstructive techniques not compared directly.
63	Christopoulos et al., 2016 [S63]	Greece	Retrospective study (1,795 patients)	Nasal cutaneous malignancies (87.7% BCC)	Direct closure, local flaps, and skin grafts following nasal tumor excision	Tumor histology, anatomical subsite, reconstructive technique, recurrence predictors	Recurrence, surgical management, prognostic factors	Direct closure was most frequently used, while local flaps and grafts were preferred for larger, high-risk defects. Columellar location and SCC histology were associated with increased recurrence.	Retrospective design; limited reporting of functional and aesthetic outcomes; no direct comparison between reconstructive techniques.
64	Khoury et al., 2026 [S64]	Canada	Retrospective study (108 patients)	Nasal BCC/SCC	Nasal reconstruction with paramedian forehead flap; donor-site closure by primary closure, skin graft, or hemicoronal flap	Defect size, donor-site closure technique	Scar quality (POSAS), patient satisfaction, complications	Hemicoronal flap achieved the best scar quality and patient satisfaction with no complications, while primary closure showed higher tension-related complications in medium-sized defects.	Retrospective single-surgeon study; mixed histology (BCC and SCC); focuses on donor-site outcomes rather than the reconstructed nasal defect.
65	Rudolph et al., 2019 [S65]	USA	Retrospective study (53 patients)	Nasal malignancies (69.8% BCC)	Cross-paramedian forehead flap for large and complex nasal defects	Diabetes, smoking, defect size, cartilage loss, mucosal loss, periorbital extension	Flap survival, complications, revision surgery	Cross-paramedian forehead flap provided reliable reconstruction of extensive nasal defects. Diabetes, cartilage defects, and mucosal defects were independently associated with significantly higher complication rates.	Retrospective single-center study; mixed tumor histology; no direct comparison with alternative reconstructive techniques.
66	Lemaître et al., 2018 [S66]	France	Retrospective case series (n = 25)	Lower eyelid malignant tumors (17 BCC, 3 spindle cell carcinoma, 5 conjunctival melanoma)	Full-thickness lower eyelid defects reconstructed using a nasal chondromucosal graft and an upper eyelid myocutaneous flap	Tumor histology, surgical margins	Functional outcome, aesthetic outcome, recurrence, postoperative complications	Reconstruction achieved good functional and aesthetic outcomes. Only one local recurrence (BCC) occurred during follow-up. No lagophthalmos or ocular surface complications were observed, and only minor eyelid retraction/scleral show occurred in a few patients.	Retrospective single-center case series; mixed tumor population; no comparison group; reconstructive outcomes not analyzed separately for BCC.

No.	Author, year	Country	Design/sample	Tumor/site	Reconstruction	Predictors	Outcomes	Key results	Limitations
67	Ghassemi et al., 2017 [S67]	Germany	Retrospective case series (n = 21)	Basal cell carcinoma of the lower third of the nose	Full-thickness nasal defects reconstructed using a forehead flap, reversed nasolabial flap, and auricular cartilage graft	Full-thickness lower nasal defects requiring three-layer reconstruction	Functional outcome, aesthetic outcome, postoperative complications, revision surgery	All patients underwent successful reconstruction and reported satisfactory functional and aesthetic outcomes. Three patients required secondary revision for contour irregularities, and one patient developed wound infection requiring repeat cartilage grafting. Donor-site morbidity was minimal.	Retrospective single-center case series; no comparison group; relatively small sample size; outcomes not assessed using validated patient-reported measures.
68	Albanese et al., 2020 [S68]	United Kingdom	Retrospective case series (n = 42)	Periocular basal cell carcinoma involving the medial lower eyelid, nasal sidewall, and infraorbital cheek	Defects reconstructed using a modified cheek advancement flap following Mohs micrographic surgery	Risk factors for flap failure, reconstructive technique	Early and late complications, ectropion, additional reconstructive procedures, patient satisfaction	Early complications occurred in 11.9% of patients and resolved within 4 weeks. No long-term complications or postoperative ectropion were observed. Overall cosmetic outcomes were excellent, with 78.6% of patients reporting they were extremely satisfied.	Retrospective single-surgeon case series; no comparison group; single-center study.
69	McKelvie et al., 2017 [S69]	New Zealand	Retrospective consecutive case series (n = 122)	Lower eyelid malignant lesions, predominantly basal cell carcinoma (>80%)	Lower eyelid defects reconstructed using Hughes tarsoconjunctival advancement flap, with additional reconstructive procedures when required	Defect size, flap width, sex, timing of flap division, need for adjuvant reconstruction	Functional outcome, cosmetic outcome, postoperative complications, revision surgery, patient satisfaction	Hughes flap reconstruction provided excellent functional and cosmetic outcomes across a wide range of lower eyelid defects. Overall complication rate was 14%, with revision surgery required in 4% of cases. Earlier flap division was associated with more complicated cases.	Retrospective study; mixed tumor population (although >80% BCC); single-country experience; mean follow-up of 7 months.
70	Jović et al., 2016 [S70]	Serbia	Retrospective case series (n = 44)	Basal cell carcinoma of the nose	Nasal skin defects reconstructed with local flaps or full-thickness skin grafts according to nasal subunit	Nasal subunit (tip, ala, sidewall, dorsum), reconstructive method	Functional outcome, aesthetic outcome	Different reconstructive techniques produced different functional and aesthetic results within the same nasal subunit, and the same technique yielded different outcomes depending on the nasal subunit involved. Appropriate reconstruction should therefore be tailored to defect location.	Retrospective single-center study; relatively small cohort; no comparison group; limited quantitative reporting of functional and aesthetic outcomes.
71	Ribeiro et al., 2022 [S71]	Brazil	Retrospective cross-sectional study; 111 patients	Predominantly BCC (NMSC cohort), nose (dorsum and tip most frequently affected)	Extensive nasal defects reconstructed with paramedian forehead flap (PMFF); lining and structural framework reconstructed in ~50% of cases; donor site mainly healed by secondary intention	Tumor stage (T1/T2 predominance), histology, nasal subunit involvement, reconstructive components	Complication rate, timing of pedicle division, time to complete reconstruction, safety and effectiveness	PMFF was a safe and effective option for complex nasal NMSC defects, with low complication rates; pedicle division was usually performed at the second stage; median time to complete reconstruction was 6 months	Retrospective, single-center study; mixed NMSC cohort (although BCC was the predominant histology); no BCC-only subgroup analysis; no comparative reconstruction group.
72	Chen et al., 2023 [S72]	China	Retrospective case series; 43 patients	BCC (26/43), SCC (8), ACC (3), MEC (3), verrucous carcinoma (2), melanoma (1); orbital, infraorbital, glabellar, nasal, cheek, upper lip, palate, lower gingiva	Oral and maxillofacial defects reconstructed with facial-angular artery island flaps (FAAIF, n=14) or reverse-flow FAAIF (R-FAAIF, n=29)	Flap type (FAAIF vs. R-FAAIF), tumor stage, defect location	Flap survival, complications, ectropion, limitation of mouth opening, aesthetic outcome, oncologic follow-up, survival	Flap success rate 97.7% (1 flap failure); complications in 34.9%; mouth opening limitation/ectropion in 28.0%; 83.7% achieved satisfactory aesthetic outcomes; no significant difference in aesthetic results or survival between FAAIF and R-FAAIF groups	Retrospective, single-center study; mixed tumor cohort (although BCC was the predominant histology); outcomes not analyzed separately for BCC; included oral and maxillofacial defects beyond exclusively facial cutaneous BCC.

Note. The 72-row curated matrix used for the secondary analysis. The tumor/site field supplies the histologic and anatomical description used for eligibility review; each author–year label is linked to Supplementary References S1–S72 and to the main-reference crosswalk in Table S6.

Table S2. Revised operational coding framework

Variable	Operational definition	Notes
**Prospective design**	Design field explicitly described the study as prospective, prospective observational, prospective interventional, or prospective longitudinal.	No inference from follow-up alone.
**Comparative design**	Explicit comparison, matched design, case-control framework, or comparison of two reconstructive strategies.	Descriptive multi-technique series were not automatically comparative.
**BCC-only**	Study population restricted to basal cell carcinoma.	Cohorts containing SCC, melanoma, Merkel cell carcinoma, or broader NMSC were coded as mixed.
**Local/locoregional flap**	Local advancement, rotation, transposition, island, perforator, forehead, nasolabial, glabellar, bilobed, Hughes, Tenzel, Mustardé, or related flap.	Non-mutually exclusive with grafts or composite support.
Any patient-reported endpoint	An outcome explicitly obtained from patients, including validated PROM scores or nonvalidated satisfaction assessments.	A clinician statement that patients were satisfied was insufficient unless the matrix attributed the assessment to patients.
Any named standardized instrument	POSAS, VSS, FACE-Q, Skin Cancer Index, or another named standardized instrument.	Broad category; not synonymous with PROM use.
Validated PROM	A validated instrument completed by patients, including FACE-Q, Skin Cancer Index, or the patient component of POSAS.	Primary patient-centered measurement outcome.
Nonvalidated patient satisfaction	An ad hoc or unspecified patient satisfaction/aesthetic rating without a validated PROM.	Kept separate from validated PROM use.
**Sample size**	Patient count preferred; otherwise lesions, tumors, cases, or excisions.	Aggregate denominator is reported analytic units.
**Limitation frequencies**	Coded from explicit limitations in the narrative matrix.	Minimum frequency; not a formal risk-of-bias assessment.
Validated observer-rated measure	A standardized clinician/observer scale, including VSS.	Not a PRO or PROM.
Mixed patient/observer instrument	An instrument containing distinct patient and observer components, including POSAS.	Contributes to validated PROM and observer categories where both components were documented.

Variable	Operational definition	Notes
Documentation breadth	Count of five domains documented in the matrix: oncologic, complications, aesthetic, functional, and patient-reported.	‘Absent’ means not documented in the matrix summary.

Note. Definitions used to convert narrative matrix summaries into structured study-level variables. ‘Not documented’ refers to the matrix summary and is not a full-text absence claim.

Table S3. Detailed exploratory analyses

Analysis	N	Any patient-reported	Validated PROM	Named standardized	≥4 domains	Exploratory ORs (PRO; PROM)
Overall curated corpus	72	9 (12.5%)	4 (5.6%)	5 (6.9%)	8 (11.1%)	PRO 9.28, p=0.011; PROM 31.00, p=0.005
Excluding database record	71	9 (12.7%)	4 (5.6%)	5 (7.0%)	8 (11.3%)	PRO 9.12, p=0.012; PROM 30.50, p=0.005
BCC-only stratum	40	4 (10.0%)	1 (2.5%)	2 (5.0%)	2 (5.0%)	PRO 3.67, p=0.355; PROM not estimable, p=0.100
Mixed-histology/NMSC	32	5 (15.6%)	3 (9.4%)	3 (9.4%)	6 (18.8%)	PRO 18.75, p=0.018; PROM 17.33, p=0.056
Issue years 2015–2020	33	4 (12.1%)	1 (3.0%)	1 (3.0%)	4 (12.1%)	PRO 9.33, p=0.231; PROM not estimable, p=0.061
Issue years 2021–2026	39	5 (12.8%)	3 (7.7%)	4 (10.3%)	4 (10.3%)	PRO 11.25, p=0.032; PROM 12.40, p=0.077

Note. Exploratory analyses for patient-reported endpoints, validated PROMs, broader named standardized measures, and documentation breadth. Sparse unadjusted odds ratios are descriptive and noncausal.

Table S4. Documentation-breadth threshold analysis

Threshold	Studies meeting threshold	Percentage
**>=3 of 5 domains**	31	43.1%
**>=4 of 5 domains**	8	11.1%
**>=5 of 5 domains**	0	0.0%

Note. Matrix documentation frequencies at thresholds of at least three, four, or all five outcome domains.

Figure S1. Limitations explicitly recorded in the narrative matrix

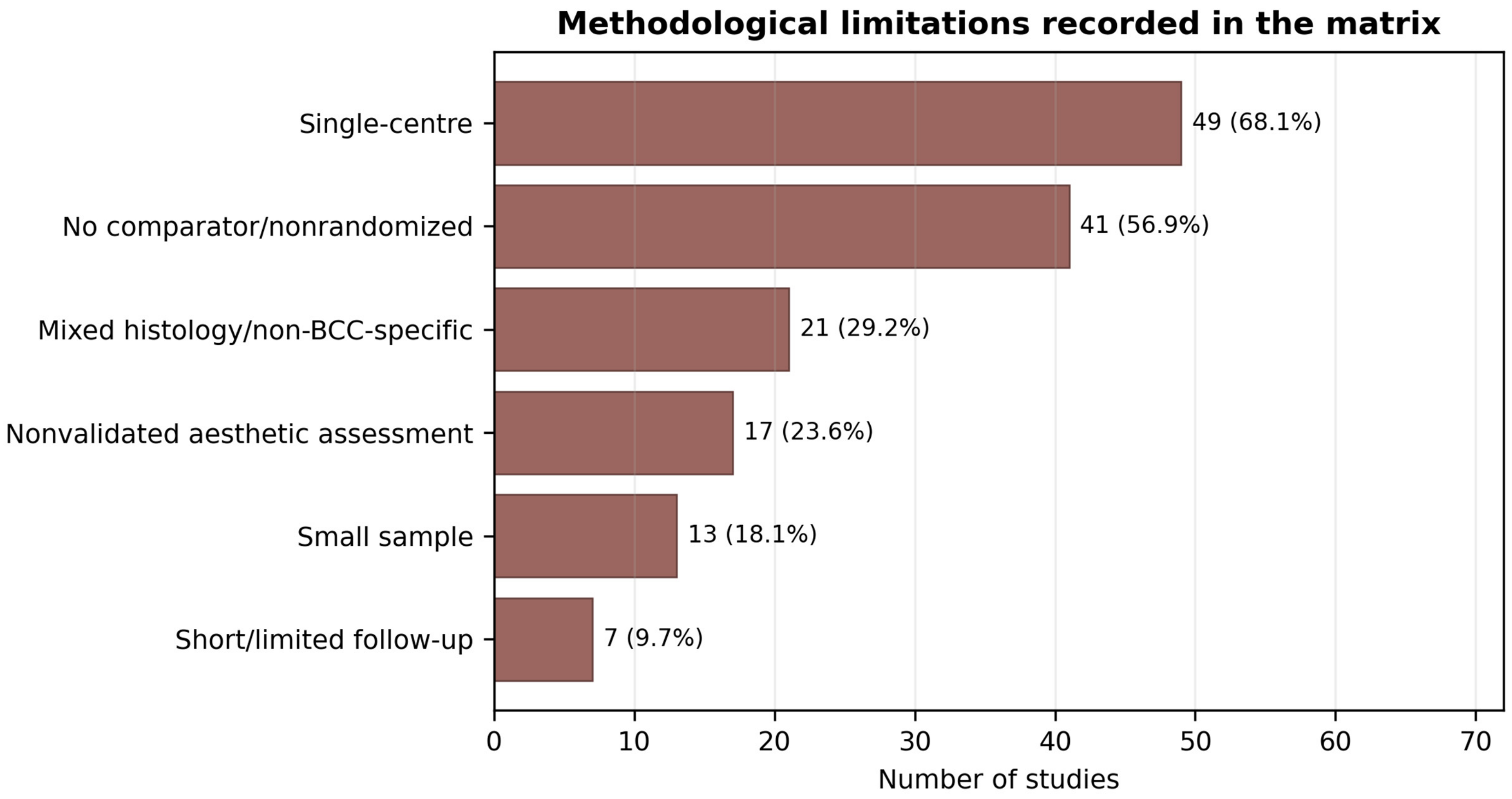

Note. Frequencies are non-mutually exclusive minimum counts and are not full-text risk-of-bias judgments.

Table S5. STROBE-oriented reporting map

Reporting element	Location/content in manuscript
Title/abstract	Title and abstract identify an exploratory analysis of a curated corpus and state the non-systematic, summary-level provenance.
**Background/objectives**	Scientific rationale, reporting gap, objectives, and hypothesis stated.
**Study design**	Study-level cross-sectional design described at the beginning of Methods.
Setting/data source	Frozen 72-row narrative matrix, issue-year range, and 31 July 2026 analytic cutoff reported; unavailable search/screening details stated.
Variables	Design, histology, anatomy, modalities, outcome domains, and PRO/PROM taxonomy operationalized.
Bias	Single-coder summary-level extraction, no full-text duplicate verification, sparse events, denominator heterogeneity, and nonexhaustive coverage described.
**Study size**	All nonempty records in the supplied matrix included; sample-size handling specified.
**Quantitative variables**	Counts, percentages, median/IQR, reporting breadth, and threshold definitions specified.
Statistical methods	Unadjusted Fisher tests, odds ratios, exact binomial confidence intervals in Figure 4, and descriptive sensitivity analyses reported.
**Participants/studies**	Corpus size, years, geography, design, histology, anatomy, and denominators reported.
**Descriptive and outcome data**	Main tables and figures report modalities, domains, associations, and sensitivity analyses.
Limitations/generalizability	All claims restricted to the curated corpus; no systematic review, prevalence, causal, or comparative effectiveness claim.
Funding/ethics/data	Funding, conflicts, ethics, contributions, AI disclosure, matrix, codebook, and bibliographic reconciliation reported.

Note. Location of applicable STROBE-oriented reporting elements in the revised exploratory meta-research manuscript.

Table S6. Record-level corpus reconciliation

Every analytic row was matched to its supplementary and corrected main manuscript reference. Histologic and anatomical descriptions are presented in Table S1. All rows contribute to the overall study-level description and to the BCC-only or mixed-histology sensitivity stratum encoded in the analytic dataset.

Row / S-ref	Study	Corrected main ref	Bibliographic and eligibility audit	Analytic contribution
1 / S1	Thakurani et al., 2022	[18]	Exact match verified; eligible within frozen matrix criteria	Included in overall analysis and histologic-scope sensitivity analysis
2 / S2	Mishra et al., 2024	[19]	Exact match verified; eligible within frozen matrix criteria	Included in overall analysis and histologic-scope sensitivity analysis
3 / S3	Dhar et al., 2026	[20]	Exact match verified; eligible within frozen matrix criteria	Included in overall analysis and histologic-scope sensitivity analysis
4 / S4	Maimaiti et al., 2016	[21]	Exact match verified; eligible within frozen matrix criteria	Included in overall analysis and histologic-scope sensitivity analysis
5 / S5	Tahir et al., 2024	[22]	Exact match verified; eligible within frozen matrix criteria	Included in overall analysis and histologic-scope sensitivity analysis
6 / S6	Zhang et al., 2020	[23]	Exact match verified; eligible within frozen matrix criteria	Included in overall analysis and histologic-scope sensitivity analysis
7 / S7	Bejinariu et al., 2020	[24]	Exact match verified; eligible within frozen matrix criteria	Included in overall analysis and histologic-scope sensitivity analysis

Row / S-ref	Study	Corrected main ref	Bibliographic and eligibility audit	Analytic contribution
8 / S8	Zgolli et al., 2023	[25]	Exact match verified; eligible within frozen matrix criteria	Included in overall analysis and histologic-scope sensitivity analysis
9 / S9	Nishimura et al., 2026	[26]	Exact match verified; eligible within frozen matrix criteria	Included in overall analysis and histologic-scope sensitivity analysis
10 / S10	McCabe et al., 2024	[27]	Exact match verified; eligible within frozen matrix criteria	Included in overall analysis and histologic-scope sensitivity analysis
11 / S11	Ikoma et al., 2025	[28]	Exact match verified; eligible within frozen matrix criteria	Included in overall analysis and histologic-scope sensitivity analysis
12 / S12	Pupić-Bakrač et al., 2025	[29]	Exact match verified; eligible within frozen matrix criteria	Included in overall analysis and histologic-scope sensitivity analysis
13 / S13	Wu et al., 2018	[30]	Exact match verified; eligible within frozen matrix criteria	Included in overall analysis and histologic-scope sensitivity analysis
14 / S14	Ofaiche et al., 2016	[6]	Exact match verified; eligible within frozen matrix criteria	Included in overall analysis and histologic-scope sensitivity analysis
15 / S15	Ceder et al., 2021	[5]	Exact match verified; eligible within frozen matrix criteria	Included in overall analysis and histologic-scope sensitivity analysis

Row / S-ref	Study	Corrected main ref	Bibliographic and eligibility audit	Analytic contribution
16 / S16	Dölen et al., 2018	[31]	Exact match verified; eligible within frozen matrix criteria	Included in overall analysis and histologic-scope sensitivity analysis
17 / S17	Derebaşınlıoğlu and Özkaya, 2021	[32]	Exact match verified; eligible within frozen matrix criteria	Included in overall analysis and histologic-scope sensitivity analysis
18 / S18	Unlu et al., 2024	[33]	Exact match verified; eligible within frozen matrix criteria	Included in overall analysis and histologic-scope sensitivity analysis
19 / S19	Aristokleous et al., 2022	[34]	Exact match verified; eligible within frozen matrix criteria	Included in overall analysis and histologic-scope sensitivity analysis
20 / S20	van Winden et al., 2022	[35]	Exact match verified; eligible within frozen matrix criteria	Included in overall analysis and histologic-scope sensitivity analysis
21 / S21	Balchev et al., 2022	[36]	Exact match verified; eligible within frozen matrix criteria	Included in overall analysis and histologic-scope sensitivity analysis
22 / S22	Tamaș et al., 2018	[37]	Exact match verified; eligible within frozen matrix criteria	Included in overall analysis and histologic-scope sensitivity analysis
23 / S23	van Kester et al., 2019	[4]	Exact match verified; eligible within frozen matrix criteria	Included in overall analysis and histologic-scope sensitivity analysis

Row / S-ref	Study	Corrected main ref	Bibliographic and eligibility audit	Analytic contribution
24 / S24	Egeler et al., 2019	[38]	Exact match verified; eligible within frozen matrix criteria	Included in overall analysis and histologic-scope sensitivity analysis
25 / S25	Gniadecki et al., 2015	[3]	Exact match verified; eligible within frozen matrix criteria	Included in overall analysis and histologic-scope sensitivity analysis
26 / S26	Balchev, 2024	[39]	Exact match verified; eligible within frozen matrix criteria	Included in overall analysis and histologic-scope sensitivity analysis
27 / S27	Chen et al., 2025	[40]	Exact match verified; eligible within frozen matrix criteria	Included in overall analysis and histologic-scope sensitivity analysis
28 / S28	Toman et al., 2022	[41]	Exact match verified; eligible within frozen matrix criteria	Included in overall analysis and histologic-scope sensitivity analysis
29 / S29	Mathieu et al., 2024	[42]	Exact match verified; eligible within frozen matrix criteria	Included in overall analysis and histologic-scope sensitivity analysis
30 / S30	Mutlu et al., 2016	[43]	Exact match verified; eligible within frozen matrix criteria	Included in overall analysis and histologic-scope sensitivity analysis
31 / S31	Marcasciano et al., 2017	[44]	Exact match verified; eligible within frozen matrix criteria	Included in overall analysis and histologic-scope sensitivity analysis

Row / S-ref	Study	Corrected main ref	Bibliographic and eligibility audit	Analytic contribution
32 / S32	DeWitt and Holmes, 2019	[45]	Exact match verified; eligible within frozen matrix criteria	Included in overall analysis and histologic-scope sensitivity analysis
33 / S33	van Hensbergen et al., 2022	[46]	Exact match verified; eligible within frozen matrix criteria	Included in overall analysis and histologic-scope sensitivity analysis
34 / S34	Zlatarova et al., 2016	[47]	Exact match verified; eligible within frozen matrix criteria	Included in overall analysis and histologic-scope sensitivity analysis
35 / S35	Romzova et al., 2024	[48]	Exact match verified; eligible within frozen matrix criteria	Included in overall analysis and histologic-scope sensitivity analysis
36 / S36	Jung et al., 2024	[49]	Exact match verified; eligible within frozen matrix criteria	Included in overall analysis and histologic-scope sensitivity analysis
37 / S37	Garg et al., 2025	[50]	Exact match verified; eligible within frozen matrix criteria	Included in overall analysis and histologic-scope sensitivity analysis
38 / S38	Chen et al., 2026	[51]	Exact match verified; eligible within frozen matrix criteria	Included in overall analysis and histologic-scope sensitivity analysis
39 / S39	Grieco et al., 2018	[52]	Exact match verified; eligible within frozen matrix criteria	Included in overall analysis and histologic-scope sensitivity analysis

Row / S-ref	Study	Corrected main ref	Bibliographic and eligibility audit	Analytic contribution
40 / S40	Uzun et al., 2015	[53]	Exact match verified; eligible within frozen matrix criteria	Included in overall analysis and histologic-scope sensitivity analysis
41 / S41	Kofler et al., 2022	[54]	Exact match verified; eligible within frozen matrix criteria	Included in overall analysis and histologic-scope sensitivity analysis
42 / S42	Galindo-Ferreiro et al., 2023	[55]	Exact match verified; eligible within frozen matrix criteria	Included in overall analysis and histologic-scope sensitivity analysis
43 / S43	Gu et al., 2026	[56]	Exact match verified; eligible within frozen matrix criteria	Included in overall analysis and histologic-scope sensitivity analysis
44 / S44	Kinde et al., 2024	[57]	Exact match verified; eligible within frozen matrix criteria	Included in overall analysis and histologic-scope sensitivity analysis
45 / S45	Bascón Rodríguez et al., 2025	[58]	Exact match verified; eligible within frozen matrix criteria	Included in overall analysis and histologic-scope sensitivity analysis
46 / S46	Aggarwal et al., 2018	[59]	Exact match verified; eligible within frozen matrix criteria	Included in overall analysis and histologic-scope sensitivity analysis
47 / S47	Neuner et al., 2021	[60]	Exact match verified; eligible within frozen matrix criteria	Included in overall analysis and histologic-scope sensitivity analysis

Row / S-ref	Study	Corrected main ref	Bibliographic and eligibility audit	Analytic contribution
48 / S48	Abdelmofeed, 2022	[61]	Exact match verified; eligible within frozen matrix criteria	Included in overall analysis and histologic-scope sensitivity analysis
49 / S49	Altin Ekin & Karadeniz Ugurlu, 2019	[62]	Exact match verified; eligible within frozen matrix criteria	Included in overall analysis and histologic-scope sensitivity analysis
50 / S50	Brandão et al., 2020	[63]	Exact match verified; eligible within frozen matrix criteria	Included in overall analysis and histologic-scope sensitivity analysis
51 / S51	Fabrizio, 2015	[64]	Exact match verified; eligible within frozen matrix criteria	Included in overall analysis and histologic-scope sensitivity analysis
52 / S52	Steele et al., 2021	[65]	Exact match verified; eligible within frozen matrix criteria	Included in overall analysis and histologic-scope sensitivity analysis
53 / S53	Rickstrew et al., 2024	[66]	Exact match verified; eligible within frozen matrix criteria	Included in overall analysis and histologic-scope sensitivity analysis
54 / S54	La Padula et al., 2017	[67]	Exact match verified; eligible within frozen matrix criteria	Included in overall analysis and histologic-scope sensitivity analysis
55 / S55	Goldman & Wollina, 2020	[68]	Exact match verified; eligible within frozen matrix criteria	Included in overall analysis and histologic-scope sensitivity analysis

Row / S-ref	Study	Corrected main ref	Bibliographic and eligibility audit	Analytic contribution
56 / S56	Rougier et al., 2019	[69]	Exact match verified; eligible within frozen matrix criteria	Included in overall analysis and histologic-scope sensitivity analysis
57 / S57	Altin Ekin & Ugurlu, 2020	[70]	Exact match verified; eligible within frozen matrix criteria	Included in overall analysis and histologic-scope sensitivity analysis
58 / S58	Lindsay & Morton, 2015	[71]	Exact match verified; eligible within frozen matrix criteria	Included in overall analysis and histologic-scope sensitivity analysis
59 / S59	Inozu et al., 2016	[72]	Exact match verified; eligible within frozen matrix criteria	Included in overall analysis and histologic-scope sensitivity analysis
60 / S60	Almubarak et al., 2026	[73]	Exact match verified; eligible within frozen matrix criteria	Included in overall analysis and histologic-scope sensitivity analysis
61 / S61	Kimura et al., 2026	[74]	Exact match verified; eligible within frozen matrix criteria	Included in overall analysis and histologic-scope sensitivity analysis
62 / S62	Al Harthi et al., 2025	[75]	Exact match verified; eligible within frozen matrix criteria	Included in overall analysis and histologic-scope sensitivity analysis
63 / S63	Christopoulos et al., 2016	[76]	Exact match verified; eligible within frozen matrix criteria	Included in overall analysis and histologic-scope sensitivity analysis

Row / S-ref	Study	Corrected main ref	Bibliographic and eligibility audit	Analytic contribution
64 / S64	Khoury et al., 2026	[77]	Exact match verified; eligible within frozen matrix criteria	Included in overall analysis and histologic-scope sensitivity analysis
65 / S65	Rudolph et al., 2019	[78]	Exact match verified; eligible within frozen matrix criteria	Included in overall analysis and histologic-scope sensitivity analysis
66 / S66	Lemaître et al., 2018	[79]	Exact match verified; eligible within frozen matrix criteria	Included in overall analysis and histologic-scope sensitivity analysis
67 / S67	Ghassemi et al., 2017	[80]	Exact match verified; eligible within frozen matrix criteria	Included in overall analysis and histologic-scope sensitivity analysis
68 / S68	Albanese et al., 2020	[81]	Exact match verified; eligible within frozen matrix criteria	Included in overall analysis and histologic-scope sensitivity analysis
69 / S69	McKelvie et al., 2017	[82]	Exact match verified; eligible within frozen matrix criteria	Included in overall analysis and histologic-scope sensitivity analysis
70 / S70	Jović et al., 2016	[83]	Exact match verified; eligible within frozen matrix criteria	Included in overall analysis and histologic-scope sensitivity analysis
71 / S71	Ribeiro et al., 2022	[84]	Exact match verified; eligible within frozen matrix criteria	Included in overall analysis and histologic-scope sensitivity analysis

Row / S-ref	Study	Corrected main ref	Bibliographic and eligibility audit	Analytic contribution
72 / S72	Chen et al., 2023	[85]	Exact match verified; eligible within frozen matrix criteria	Included in overall analysis and histologic-scope sensitivity analysis

Table S7. Screening of the reviewer-nominated study

The reviewer-nominated article was screened explicitly. It is clinically relevant but was not added post hoc to the frozen quantitative corpus because the parent matrix was not generated by a reproducible search; adding one known record would create a selectively augmented denominator without resolving incompleteness.

Study	Clinical topic	Screening result	Quantitative corpus status	Reason
Gąsiorowski et al. J. Clin. Med. 2024;13:6120. doi:10.3390/jcm13206120	Reconstruction after malignant eyelid tumor excision; mixed histology including BCC	Relevant to the clinical scope	Not included	Outside the frozen parent matrix; selective post hoc addition would not produce a systematic or reproducible denominator. The record demonstrates nonexhaustiveness and supports the revised scope restriction.

Supplementary References S1–S72

The following references correspond one-to-one with the 72 reports in Supplementary Table S1. Table S6 records each report's corrected main-manuscript number. The six unrelated bibliography entries identified during peer review are not part of this list or the analytic matrix.

S1. Thakurani, S.; Gupta, S.; Mohammad, A.; Escandón, J.M. Facial Basal Cell Carcinoma: A Study of Causative Factors and Site-Based Algorithm for Surgical Reconstruction. J. Cutan. Aesthet. Surg. 2022, 15, 275–283. https://doi.org/10.4103/JCAS.JCAS_113_21.

S2. Mishra, B.; Mallik, S.; Agnihotry, I.; Behera, J. Aesthetic Reconstruction Based on Facial Subunit Principle for Basal Cell Carcinoma of the Face: A Retrospective Analysis. Cureus 2024, 16, e56826. https://doi.org/10.7759/cureus.56826.

S3. Dhar, L.K.; Ghosh, T.P.; Jahan, I.; Razia, S.; Talukder, A.; Hossain, M.A.; Bithi, D.D.; Kaiser, A. Facial Basal Cell Carcinoma: Clinical Profile, Surgical Management and Outcomes. Mymensingh Med. J. 2026, 35, 420–426.

S4. Maimaiti, A.; Mijiti, A.; Yarbag, A.; Moming, A. Giant Basal Cell Carcinoma of the Face: Surgical Management and Challenges for Reconstruction. J. Laryngol. Otol. 2016, 130, 176–182. https://doi.org/10.1017/S0022215115003333.

S5. Tahir, M.; Bashir, U.; Akhtar, A.; Javaid, S.; Ansari, Z.; Ansari, S. Basal Cell Carcinoma Excision Intraoperative Frozen Section for Tumor Clearance Rate and Reconstructive Surgery. J. Ayub Med. Coll. Abbottabad 2024, 36, 692–697. https://doi.org/10.55519/JAMC-04-13800.

S6. Zhang, J.; Lu, Y.; Zhang, X.; Yang, Y.; Kou, H.; Wang, Y. Clinical Efficacy of Mohs Surgery Combined with Topical Photodynamic Therapy for Facial Basal Cell Carcinoma. J. Cancer Res. Ther. 2020, 16, 1051–1055. https://doi.org/10.4103/jcrt.JCRT_987_19.

S7. Bejinariu, C.G.; Popescu, S.; Dragosloveanu, C.D.M.; Marinescu, S.A. Reconstruction of Lower Eyelid Defects after the Excision of Basal Cell Carcinoma. Rom. J. Ophthalmol. 2020, 64, 414–418. https://doi.org/10.22336/rjo.2020.64.

S8. Zgolli, H.; Choura, R.; Elzarrug, H.K.H.; Abdelhedi, C.; Chebbi, A. Management of Eyelids' Primary Malignancies. Libyan J. Med. 2023, 18, 2258668. https://doi.org/10.1080/19932820.2023.2258668.

S9. Nishimura, K.; Kimura, A.; Nakamura, A.; Takatsuka, S.; Takenouchi, T. Tumor-Related Determinants of Reconstructive Method After Excision of Facial Basal Cell Carcinoma. J. Dermatol. 2026, 53, 943–947. https://doi.org/10.1111/1346-8138.70248.

S10. McCabe, G.A.; Mulcahy, L.T.; Mulligan, N.; Fulcher, T. Recurrence Rates of Periocular Basal Cell Carcinoma Following Intra-Operative En-Face Frozen Section Margin Controlled Excision. Ir. J. Med. Sci. 2024, 193, 1209–1213. https://doi.org/10.1007/s11845-024-03620-w.

S11. Ikoma, T.; Amagata, Y.; Fujii, K.; Aoki, M.; Tozawa, T.; Iwata, M.; Yamamura, K.; Hirano, Y.; Sakamoto, S.; Matsushita, S. Outcomes of Free Tarsoconjunctival Grafts in Lower Eyelid Reconstruction Following Basal Cell Carcinoma Surgery. J. Dermatol. 2025, 52, 1465–1469. https://doi.org/10.1111/1346-8138.17872.

S12. Pupić-Bakrač, J.; Pupić-Bakrač, A.; Matoc, L.; Knežević, J. Surgical Management of Giant Basal Cell Carcinoma in the Maxillofacial Region: Ablative and Reconstructive Strategies. Ann. Plast. Surg. 2025, 94, 312–319. https://doi.org/10.1097/SAP.0000000000004227.

S13. Wu, J.; Qing, Y.; Cen, Y.; Chen, J. Frontal Axial Pattern Flap Combined with Hard Palate Mucosa Transplant in the Reconstruction of Midfacial Defects after the Excision of Huge Basal Cell Carcinoma. World J. Surg. Oncol. 2018, 16, 120. https://doi.org/10.1186/s12957-018-1421-7.

S14. Ofaiche, J.; Lopez, R.; Bérard, E.; André, A.; Bulai-Livideanu, C.; Méresse, T.; Vairel, B.B.; Grolleau, J.L.; Paul, C.; Meyer, N. Surgical Treatment of Facial Basal Cell Carcinoma: Patient-Based Assessment of Clinical Outcome in a Prospective Cohort Study. Dermatology 2016, 232, 550–557. https://doi.org/10.1159/000447354.

S15. Ceder, H.; Grönberg, M.; Paoli, J. Mohs Micrographic Surgery for Primary Versus Recurrent or Incompletely Excised Facial High-Risk Basal Cell Carcinomas. Acta Derm. Venereol. 2021, 101, adv00381. https://doi.org/10.2340/00015555-3698.

S16. Dölen, U.C.; Baltu, Y.; Aydin, O. Subunit Reconstruction of Mid-Facial Defects with Free Style Facial Perforator Flaps. J. Craniofac. Surg. 2018, 29, 1574–1577. https://doi.org/10.1097/SCS.0000000000004535.

S17. Derebaşınlıoğlu, H.; Özkaya, N.K. Analysis of Basal Cell Carcinoma and Squamous Cell Carcinoma According to Nasal Subunit Location. Facial Plast. Surg. 2021, 37, 407–410. https://doi.org/10.1055/s-0041-1726024.

S18. Unlu, M.; Ozer, F.; Vural, A. A Comparison of Two Surgical Techniques for Basal Cell Carcinoma of the Eyelid: Two-Step Surgery and One-Step Surgery. Int. Ophthalmol. 2024, 45, 6. https://doi.org/10.1007/s10792-024-03379-0.

S19. Aristokleous, I.; Schultz, I.; Vassilaki, I.; Krynitz, B.; Lapins, J.; Girnita, A.; Nilsson, M.F. Mohs Micrographic Surgery Revisited: A Multidisciplinary, Collaborative Approach for the Treatment of Aggressive and Recurrent Basal Cell Carcinoma on the Head and Neck. J. Plast. Reconstr. Aesthet. Surg. 2022, 75, 3373–3383. https://doi.org/10.1016/j.bjps.2022.04.037.

S20. van Winden, M.E.C.; Bronkhorst, E.M.; Visch, M.B.; Krekels, G.A.M.; van der Geer, S.; Damen, G.W.J.A.; Amir, A.; Aben, K.K.H.; Gerritsen, M.J.P.; van de Kerkhof, P.C.M.; de Jong, E.M.G.J.; Lubeek, S.F.K. Predictors of Surgical Treatment Burden, Outcomes, and Overall Survival in Older Adults with Basal Cell Carcinoma: Results from the Prospective, Multicenter BATOA Cohort. J. Am. Acad. Dermatol. 2022, 86, 1010–1019. https://doi.org/10.1016/j.jaad.2021.05.041.

S21. Balchev, G.; Balabanov, C.; Murgova, S. Glabellar Flap Technique in Oculoplastic Surgery. Indian J. Ophthalmol. 2022, 70, 281–286. https://doi.org/10.4103/ijo.IJO_1463_21.

S22. Tamaş, C.; Pintilie, C.T.; Atănăsoae, I.V.; Corduneanu, A.M.; Dabija, I.; Olaru, F.Ş.; Hreniuc, I.M.; Tecuceanu, A.; Munteanu, I.; Dobre, C.; Moraru, D.C.; Ianole, V.; Tamaş, I.; Costan, V.V. Surgical Reconstruction of Post-Tumoral Facial Defects. Rom. J. Morphol. Embryol. 2018, 59, 285–291.

S23. van Kester, M.S.; Goeman, J.J.; Genders, R.E. Tissue-Sparing Properties of Mohs Micrographic Surgery for Infiltrative Basal Cell Carcinoma. J. Am. Acad. Dermatol. 2019, 80, 1700–1703. https://doi.org/10.1016/j.jaad.2019.01.057.

S24. Egeler, S.A.; Johnson, A.R.; Ibrahim, A.M.S.; Bucknor, A.; Chen, A.; Malyar, M.; Tobias, A.M.; Lin, S.J.; Mureau, M.A.M.; Lee, B.T. Reconstruction of Mohs Defects Located in the Head and Neck. J. Craniofac. Surg. 2019, 30, 412–417. https://doi.org/10.1097/SCS.0000000000005137.

S25. Gniadecki, R.; Glud, M.; Mortensen, K.; Bang, B.; Biskup, E.; Omland, S.H. Favourable Results of Mohs Micrographic Surgery for Basal Cell Carcinoma. Dan. Med. J. 2015, 62, A5171.

S26. Balchev, G. Complications and Recurrences after Excision and Reconstruction of Eyelid Tumors. Curr. Oncol. 2024, 31, 1713–1724. https://doi.org/10.3390/curroncol31040130.

S27. Chen, H.; Li, Q.; Zhe, N.; Huang, N. Fine Sutures Combined with Local Flaps in the Cosmetic Repair of Surgical Defects of Basal Cell Carcinoma of the Head and Face: A Single-Center Retrospective Clinical Study. Eur. J. Med. Res. 2025, 30, 896. https://doi.org/10.1186/s40001-025-03163-5.

S28. Toman, J.; Michael, G.M.; Wisco, O.J.; Adams, J.R.; Hubbs, B.S. Mohs Defect Repair with Dehydrated Human Amnion/Chorion Membrane. Facial Plast. Surg. Aesthet. Med. 2022, 24, 48–53. https://doi.org/10.1089/fpsam.2021.0167.

S29. Mathieu, O.; Chaine, A.; Benassarou, M.; Combes, F.; Debelmas, A.; Lanciaux, S.; Bertolus, C.; Bouaoud, J. Epidemiology of Facial Skin Cancers Managed in a French Ambulatory Surgical Center. J. Stomatol. Oral Maxillofac. Surg. 2024, 125, 101726. https://doi.org/10.1016/j.jormas.2023.101726.

S30. Özkaya Mutlu, Ö.; Egemen, O.; Dilber, A.; Üsçetin, I. Aesthetic Unit-Based Reconstruction of Periorbital Defects. J. Craniofac. Surg. 2016, 27, 429–432. https://doi.org/10.1097/SCS.0000000000002359.

S31. Marcasciano, M.; Tarallo, M.; Maruccia, M.; Fanelli, B.; La Viola, G.; Casella, D.; Wals, L.S.; Ciaschi, S.; Fioramonti, P. Surgical Treatment with Locoregional Flap for the Nose. Biomed Res. Int. 2017, 2017, 9750135. https://doi.org/10.1155/2017/9750135.

S32. DeWitt, C.; Holmes, T. Reconstruction of Preauricular Wounds Using a Flipped Island Pedicle Flap. Dermatol. Surg. 2019, 45, 44–51. https://doi.org/10.1097/DSS.0000000000001610.

S33. van Hensbergen, L.J.; Veldhuizen, I.J.; Lee, E.H.; Houterman, S.; Brinkhuizen, T.; van der Hulst, R.R.W.J.; Hoogbergen, M.M. Cancer Worry after Facial Nonmelanoma Skin Cancer Resection and Reconstruction: A 1-Year Prospective Study. Psychooncology 2022, 31, 238–244. https://doi.org/10.1002/pon.5810.

S34. Zlatarova, Z.I.; Nenkova, B.N.; Softova, E.B. Eyelid Reconstruction with Full Thickness Skin Grafts after Carcinoma Excision. Folia Med. (Plovdiv) 2016, 58, 42–47. https://doi.org/10.1515/folmed-2016-0006.

S35. Romzova, B.; Matuskova, D.; Palencar, D.; Belak, J.; Knazovicky, M. Reconstructive Techniques for Lower Eyelid and Canthal Defects after Tumor Resection. Bratisl. Lek. Listy 2024, 125, 666–676. https://doi.org/10.4149/BLL_2024_102.

S36. Jung, S.; Lee, S.; Eun, S. A Novel Reconstruction Approach after Skin Cancer Ablation Using Lateral Arm Free Flap: A Serial Case Report. Medicina (Kaunas) 2024, 60, 2082. https://doi.org/10.3390/medicina60122082.

S37. Garg, N.; Vaile, J.R.; Amin, D.; Xu, V.; McCann, A.; Kumar, A.; Urdang, Z.; Krein, H.; Heffelfinger, R. Evaluating the Effects of Timing of Reconstruction of Facial Mohs Defects for Nonmelanoma Skin Cancer on Complications Using Data from a Global Aggregate Health Care Network. Facial Plast. Surg. Aesthet. Med. 2025, 27, 344–349. https://doi.org/10.1089/fpsam.2024.0215.

S38. Chen, J.; Fu, Z.; Xie, Y.; Wu, N.; Zhou, L.; Li, Y.; Wu, M.; Jinrong, Z. Application of a Subcutaneous Tissue Pedicle Flap Combined with a Labial Mucosa Flap for Reconstruction of Full-Thickness Lower Lip Defects: A Prospective Observational Study. J. Dermatolog. Treat. 2026, 37, 2617778. https://doi.org/10.1080/09546634.2026.2617778.

S39. Grieco, M.P.; Bertozzi, N.; Grignaffini, E.; Raposio, E. Nose Defects Reconstruction with the Zitelli Bilobed Flap. G. Ital. Dermatol. Venereol. 2018, 153, 278–282. https://doi.org/10.23736/S0392-0488.17.05035-0.

S40. Uzun, H.; Bitik, O.; Kamburoğlu, H.O.; Dadaci, M.; Çaliş, M.; Öcal, E. Assessment of Patients Who Underwent Nasal Reconstruction after Non-Melanoma Skin Cancer Excision. J. Craniofac. Surg. 2015, 26, 1299–1303. https://doi.org/10.1097/SCS.0000000000001563.

S41. Kofler, K.; Häfner, H.M.; Eckardt, J.; Scheu, A.; Ulmer, A.; Schulz, C.; Kofler, L. Modification of Skin Flaps for Single-Stage Reconstruction of the Nasal Ala Using All-Layer Suture: A Prospective Study. J. Cutan. Med. Surg. 2022, 26, 465–472. https://doi.org/10.1177/12034754221101362.

S42. Galindo-Ferreiro, A.; Akaishi, P.M.; Bermudez-Castellanos, I.; Cruz, A.V. Lateral Eyelid Island Flap for Reconstruction of Large Central and Medial Full-Thickness Defects of the Lower Eyelid. Eur. J. Ophthalmol. 2023, 33, 1484–1489. https://doi.org/10.1177/11206721221146817.

S43. Gu, J.; Cao, L.; Zou, Y.; Feng, G. Nasolabial Fold Retrograde Island Flap with Local Heparin Injection for Nasal Defect Repair Following Basal Cell Carcinoma Excision. J. Vis. Exp. 2026, 232, e71081. https://doi.org/10.3791/71081.

S44. Kinde, B.; Idowu, O.O.; Ashraf, D.C.; Chen, R.M.; Hirabayashi, K.E.; Grob, S.R.; Winn, B.J.; Kersten, R.C.; Vagefi, M.R. Quality-of-Life Outcomes for Excision and Reconstruction of Periocular Nonmelanoma Skin Cancer. Facial Plast. Surg. Aesthet. Med. 2024, 26, 303–309. https://doi.org/10.1089/fpsam.2020.0647.

S45. Bascón Rodríguez, L.; Iglesias Zamora, M.E.; de León Marrero, F.J.; Turrión-Merino, L.; Tejera-Vaquerizo, A.; Navarro-Guillamón, P.J.; Rodríguez-Jiménez, P.; Rodríguez Lomba, E.; Russo de la Torre, F.; García-Doval, I.; Guilabert Vidal, A.; Romaní de Gabriel, J. New Horizons for the Puzzle Flap: A Multicenter Review of 35 Cases. Actas Dermosifiliogr. 2025, 116, T1053–T1056. https://doi.org/10.1016/j.ad.2025.10.008.

S46. Aggarwal, S.; Shah, C.T.; Kirzhner, M. Modified Second Stage Hughes Tarsoconjunctival Reconstruction for Lower Eyelid Defects. Orbit 2018, 37, 335–340. https://doi.org/10.1080/01676830.2017.1423351.

S47. Neuner, R.A.; Sclafani, A.P.; Minkis, K.; Obayemi, A.; Navrazhina, K.; Cressey, B.; Ojeda, S.; Trujillo, O. Patient, Defect, and Surgical Factors Influencing Use of Ancillary Procedures after Facial Mohs Repairs. Facial Plast. Surg. 2021, 37, 390–394. https://doi.org/10.1055/s-0040-1721100.

S48. Abdelmofeed, A.M. Glabellar Superciliary Bilobed Flap for Reconstruction of Large Medial Canthus Region Defect. Br. J. Oral Maxillofac. Surg. 2022, 60, 1362–1367. https://doi.org/10.1016/j.bjoms.2022.09.004.

S49. Altin Ekin, M.; Karadeniz Ugurlu, S. Effect of Eyelid Involvement in the Reconstruction of Medial Canthal Defects. Facial Plast. Surg. 2019, 35, 410–419. https://doi.org/10.1055/s-0039-1694722.

S50. Brandão, C.M.; Weimann, E.T.S.; Terzian, L.R.; Machado Filho, C.D.S.; Paschoal, F.M.; Criado, P.R. Keep It Simple: A Ten-Year Experience in Reconstructions after Mohs Micrographic Surgery. An. Bras. Dermatol. 2020, 95, 714–720. https://doi.org/10.1016/j.abd.2020.05.004.

S51. Fabrizio, T. Indications for, and Limitations of, the Retroangular Flap in Facial Reconstruction According to Its Vascular Mapping. Br. J. Oral Maxillofac. Surg. 2015, 53, 529–532. https://doi.org/10.1016/j.bjoms.2015.03.006.

S52. Steele, T.N.; Blaha, L.E.; Madan, O.P.; Walker, N.J.; Marks, M.W. The Triangular Dart Flap: A Reconstructive Option for Soft Tissue Defects. Ann. Plast. Surg. 2021, 86, S555–S559. https://doi.org/10.1097/SAP.0000000000002697.

S53. Rickstrew, J.; Neill, B.C.; Downing, M.; Cappel, J.A.; Tolkachjov, S.N. Nasal Tip Rotation Flap for Reconstruction of Surgical Defects on the Distal Nose. Int. J. Dermatol. 2024, 63, 1421–1426. https://doi.org/10.1111/ijd.17155.

S54. La Padula, S.; Abbate, V.; Di Monta, G.; Schonauer, F. Levator Alae Nasi Muscle V-Y Island Flap for Nasal Tip Reconstruction. J. Craniomaxillofac. Surg. 2017, 45, 432–435. https://doi.org/10.1016/j.jcms.2016.12.019.

S55. Goldman, A.; Wollina, U. Defect Closure after Successful Skin Cancer Surgery of the Nose: A Report of 52 Cases. Acta Dermatovenerol. Alp. Pannonica Adriat. 2020, 29, 209–214.

S56. Rougier, G.; Meningaud, J.P.; Ganry, L.; Hermeziu, O.; Bosc, R.; Sidahmed-Mezi, M.; Hersant, B. Oncological and Aesthetic Outcome Following Surgical Management of Orbito-Palpebral Skin Cancers: A Retrospective Study of 132 Patients. J. Craniomaxillofac. Surg. 2019, 47, 1577–1582. https://doi.org/10.1016/j.jcms.2019.07.015.

S57. Ekin, M.A.; Ugurlu, S.K. Impact of the Type of Anterior Lamellar Reconstruction on the Success of Modified Hughes Procedure. Arq. Bras. Oftalmol. 2020, 83, 11–18. https://doi.org/10.5935/0004-2749.20200011.

S58. Lindsay, K.J.; Morton, J.D. Flap or Graft: The Best of Both in Nasal Ala Reconstruction. J. Plast. Reconstr. Aesthet. Surg. 2015, 68, 1352–1357. https://doi.org/10.1016/j.bjps.2015.05.036.

S59. Inozu, E.; Tellioglu, A.T.; Eryilmaz, A.T.; Ergin, S. Pincer Flap for Reconstruction of Nasal Tip Defects. J. Craniofac. Surg. 2016, 27, 769–771. https://doi.org/10.1097/SCS.0000000000002503.

S60. Almubarak, Z.; Minhas, M.H.; Khoury, S.; Demirci, D.; Moore, C.C. Reconstruction of Full-Thickness Eyelid and Periorbital Defects Using the Paramedian Forehead Flap. J. Craniofac. Surg. 2026, 37, 1953–1956. https://doi.org/10.1097/SCS.0000000000012641.

S61. Kimura, A.; Ishii, T.; Hatta, N. Efficacy and Cosmetics of Secondary Intention Healing for Facial Skin Cancer: A Single-Center Retrospective Study. J. Dermatol. 2026, 53, 1006–1012. https://doi.org/10.1111/1346-8138.70298.

S62. Al Harthi, A.S.; Park, J.; Yang, S.W. Clinical Outcomes and Prognostic Factors in Eyelid Malignancy: A 17-Year Retrospective Analysis of Surgical Management and Reconstruction Techniques. Korean J. Ophthalmol. 2025, 39, 14–22. https://doi.org/10.3341/kjo.2024.0003.

S63. Christopoulos, G.; Sergentanis, T.N.; Karantonis, F.; Karypidis, D.; Hampsas, G.; Kostopoulos, E.; Kostaki, M.; Papadopoulos, O. Surgical Treatment and Recurrence of Cutaneous Nasal Malignancies: A 26-Year Retrospective Review of 1795 Patients. Ann. Plast. Surg. 2016, 77, e2–e8. https://doi.org/10.1097/SAP.0000000000000329.

S64. Khoury, S.; Aleksa, E.; Smith, C.; Gandhi, K.; Ferreira, L.; Moore, C. Donor Site Closure in Paramedian Forehead Flap Reconstruction: Impact on Scar Quality and Patient Satisfaction. J. Craniofac. Surg. 2026, 37, 711–713. https://doi.org/10.1097/SCS.0000000000012163.

S65. Rudolph, M.A.; Walker, N.J.; Rebowe, R.E.; Marks, M.W. Broadening Applications and Insights into the Cross-Paramedian Forehead Flap over a 19-Year Period. J. Plast. Reconstr. Aesthet. Surg. 2019, 72, 763–770. https://doi.org/10.1016/j.bjps.2018.12.001.

S66. Lemaître, S.; Lévy-Gabriel, C.; Desjardins, L.; González-Candial, M.; Gardrat, S.; Dendale, R.; Cassoux, N.; Couturaud, B. Outcomes after Surgical Resection of Lower Eyelid Tumors and Reconstruction Using a Nasal Chondromucosal Graft and an Upper Eyelid Myocutaneous Flap. J. Fr. Ophtalmol. 2018, 41, 412–420. https://doi.org/10.1016/j.jfo.2017.10.008.

S67. Ghassemi, A.; Ahmed, S.S.; Ghanepur, H.; Modabber, A. Three-Layer Reconstruction of Lower Third Nasal Defects Using Forehead Flap, Reversed Nasolabial Flap, and Auricular Cartilage. Int. J. Oral Maxillofac. Surg. 2017, 46, 36–40. https://doi.org/10.1016/j.ijom.2016.08.024.

S68. Albanese, G.; Kasbekar, S.; Abercrombie, L.C. Modified Cheek Advancement Flap for Medial Lower Eyelid, Nasal Sidewall and Infraorbital Cheek Reconstruction: A Case Series. Orbit 2020, 39, 123–127. https://doi.org/10.1080/01676830.2019.1626445.

S69. McKelvie, J.; Ferguson, R.; Ng, S.G.J. Eyelid Reconstruction Using the Hughes Tarsoconjunctival Advancement Flap: Long-Term Outcomes in 122 Consecutive Cases over a 13-Year Period. Orbit 2017, 36, 228–233. https://doi.org/10.1080/01676830.2017.1310256.

S70. Jović, M.S.; Dimitrijević, M.M.; Dimitrijević, A.M.; Stojković, G.G. Analysis of Reconstructive Methods in Surgical Treatment of Nasal Skin Defects. Vojnosanit. Pregl. 2016, 73, 723–727. https://doi.org/10.2298/VSP140829052J.

S71. Ribeiro, R.D.A.; Pagotto, V.P.F.; Takahashi, G.G.; Tutihashi, R.M.C.; Camargo, C.P.; Busnardo, F.F.; Gemperli, R. Paramedian Forehead Flap in the Treatment of Nasal Non-Melanoma Skin Cancer: A Cross-Sectional Study. Rev. Col. Bras. Cir. 2022, 49, e20223034. https://doi.org/10.1590/0100-6991e-20223034-en.

S72. Chen, W.L.; Zhou, B.; Huang, Z.X.; Chen, R.; Dong, X.Y. Various Types of Facial-Angular Artery-Based Flaps for Reconstructing Oral and Maxillofacial Defects Following Cancer Ablation. J. Craniofac. Surg. 2023, 34, e478–e483. https://doi.org/10.1097/SCS.0000000000009337.
